# Cross Reactive Material 197 glycoconjugate vaccines contain privileged conjugation sites

**DOI:** 10.1038/srep20488

**Published:** 2016-02-04

**Authors:** Uwe Möginger, Anja Resemann, Christopher E. Martin, Sharavathi Parameswarappa, Subramanian Govindan, Eike-Christian Wamhoff, Felix Broecker, Detlev Suckau, Claney Lebev Pereira, Chakkumkal Anish, Peter H. Seeberger, Daniel Kolarich

**Affiliations:** 1 Department of Biomolecular Systems, Max Planck Institute of Colloids and Interfaces, 14424 Potsdam, Germany; 2 Institute of Chemistry and Biochemistry, Freie Universität Berlin, Germany; 3 Bruker, Bremen, Germany

## Abstract

Production of glycoconjugate vaccines involves the chemical conjugation of glycans to an immunogenic carrier protein such as Cross-Reactive-Material-197 (CRM_197_). Instead of using glycans from natural sources recent vaccine development has been focusing on the use of synthetically defined minimal epitopes. While the glycan is structurally defined, the attachment sites on the protein are not. Fully characterized conjugates and batch-to-batch comparisons are the key to eventually create completely defined conjugates. A variety of glycoconjugates consisting of CRM_197_ and synthetic oligosaccharide epitopes was characterised using mass spectrometry techniques. The primary structure was assessed by combining intact protein MALDI-TOF-MS, LC-MALDI-TOF-MS middle-down and LC-ESI-MS bottom-up approaches. The middle-down approach on CNBr cleaved glycopeptides provided almost complete sequence coverage, facilitating rapid batch-to-batch comparisons, resolving glycan loading and identification of side products. Regions close to the N- and C-termini were most efficiently conjugated.

Pathogens such as bacteria and parasites exhibit specific glycan structures on their surface^1^. These accessible biomolecules are targets for pathogen specific vaccination. Glycoconjugate vaccines that combine a glycan antigen with a carrier protein have for the most part replaced polysaccharide vaccines that fail to induce a strong immune response^2^ and do not result in B-cell memory in infants under the age of two^3^,^4^.

Successful and broadly marketed carbohydrate conjugate vaccines are based on just a few FDA-approved carrier proteins. First-generation carrier proteins such as diphtheria toxin and tetanus toxin require detoxification with formaldehyde eliminating part of the lysine residues needed for glycan attachment, thereby limiting conjugation efficacy^5^. One of the most widely used and highly effective carrier protein is Cross-Reactive-Material-197 (CRM_197_), a mutant version of the diphtheria toxin, where the single amino acid exchange of a glycine in position 52 to a glutamic acid renders the protein non-toxic^6^. Conjugate vaccines such as HibTITER^®^ (*Haemophilus influenzae* type b associated diseases), Prevnar*™* (pneumococcal diseases), and Menveo^®^ (meningococcal diseases)^7^ use CRM_197_ as carrier protein.

Almost all currently marketed glycoconjugate vaccines contain isolated, large and heterogeneous glycans^8^,^9^. These vaccines are very successful, but technically, the isolation from natural sources is challenging. Not all pathogenic organisms can be grown to produce antigenic carbohydrates *in vitro*. Culturing and processing large amounts of human pathogens safely is complex and cost intensive. Downstream processing inevitably produces heterogeneous glycan mixtures of various lengths and modifications that are difficult to characterise before and after conjugation. In some cases, glycan processing can affect epitope integrity^10^,^11^. Regular in-depth molecular level characterisation of the final product that will be used for immunisation is thus not possible for these biologics.

The chemical synthesis of the immunogenic glyco-epitopes^12^ holds many advantages but only one semi-synthetic glycoconjugate vaccine (Quimi Hib) is currently on the market^13^. Synthetic, minimal immunogenic glyco-epitopes are structurally well-defined and homogenous^14^. The synthetic approach enables the placement of a spacer carrying a unique functional group such as an amine to facilitate protein attachment at a single site without further activation.

Typically, glycans are coupled to the primary amine side chains of lysine residues and to the protein N-terminus via a linker molecule. Alternatively, sulfhydryl groups in cysteine or carboxyl side chains can serve as sites for attachment. Prior to coupling, non-synthetic glycans need to be activated^15^ either arbitrarily on hydroxyl or carboxyl groups^16^, leading to attachment at various points or via a single amine group, introduced by reductive amination, at the reducing end^17^. The choice of the linker is crucial since it influences vaccine properties^18^ and may be immunogenic itself^19^. In addition, coupling efficiencies have to be considered. Conjugation of a synthetic glyco-epitope to a carrier protein such as CRM_197_ that contains 40 primary amine side chains will result in heterogeneous glycoconjugates since some amino acid side chains more readily engage in conjugation reactions than others^20^. Since variations in epitope loading influence the effectiveness of vaccines^21^,^22^ understanding which side chains preferentially react with glycans represents an important step towards producing better defined, more effective and safer conjugate vaccines. Quality control and batch-to-batch comparison relies on a combination of physicochemical, spectroscopic and spectrometric methods such as nuclear magnetic resonance (NMR) or mass spectrometry (MS)^23^,^24^,^25^. Glycan attachment to the carrier protein is typically monitored by SDS-PAGE and MALDI-TOF-MS^26^. These methods provide information about average loading and cannot detect any side reactions or alterations occurring on the glycan epitopes. New methods are necessary for quality assurance of conjugate vaccines and to define conjugation sites on the protein.

We established a mass spectrometry-based assay for the in-depth characterisation of glycoconjugates obtained by coupling synthetic glycans to CRM_197_ (Fig. 1). An integrated new middle-down LC-MALDI-TOF-MS approach simplified batch-to-batch comparison, and enabled relative quantification of regional conjugation efficacy. In this study we evaluated the influence of glycan size and amino acid microenvironment on conjugation efficiencies, assessed glycan integrity and determined which amino acid sites were preferably conjugated. Two regions close to the N- and C-termini of the protein engage primarily in the chemical conjugation process and exhibited the highest loading ratios. In addition to accessibility, our data clearly illustrates that the local amino acid environment significantly influences which lysine residues will be modified.

## Results and Discussion

### Protease based bottom up strategies for the evaluation of CRM_197_ glycoconjugate vaccine candidates

Assuring glycan integrity of the glycoconjugate is important after the chemical conjugation step and to evaluate the storage stability. We studied this property by a classical (glyco)proteomics bottom up approach, which provided qualitative data on individual, repeatedly conjugated sites in CRM_197_.

For unmodified CRM_197_ the use of trypsin resulted in an average sequence coverage of 50–67%. Using Glu-C usually 50% of the entire sequence could be identified (see Supplementary Fig. S2). The highest coverage (≈70–85%) was obtained using a sequential digestion employing Glu-C followed by trypsin or Asp-N followed by Glu-C. Nevertheless, the results varied considerably from digest to digest indicating the limitation of such a bottom-up approach for routine analyses of chemically glycosylated neoglycoproteins.

Trypsin did not cleave at modified lysine residues resulting in conjugated peptides carrying at least one missed cleavage site^27^. As a result, the C-terminal lysine in any tryptic peptide had to be unmodified in order to be cleaved. This facilitated the assignment of the actual conjugation site in particular as for most glycopeptides only one lysine residue remained as a possible site of conjugation. On the other hand, the modification induced missed cleavage sites lead to an increased heterogeneity of peptide fragments and consequently reduced signal intensities.

**Figure 2:**
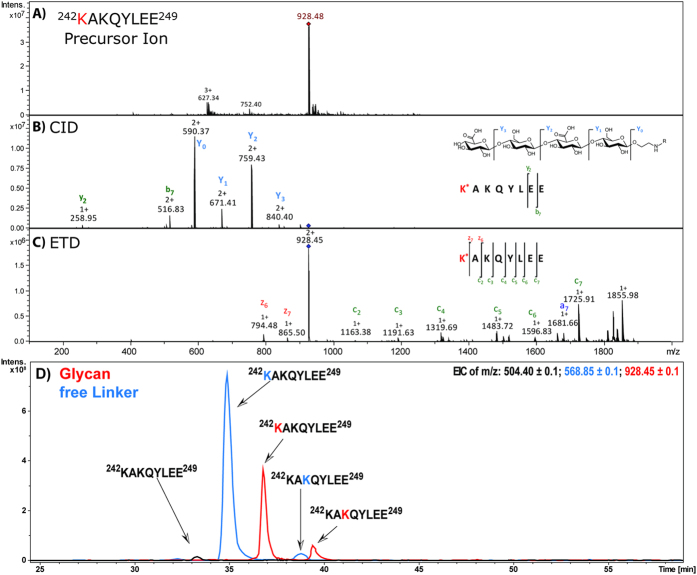
Conjugation site determination by tandem mass spectrometry. (**A**) After Glu-C digestion a doubly charged precursor ion corresponding to glycopeptide ^242^KAKQYLEE^249^ carrying an ST3 epitope was detected and selected for fragmentation by CID and ETD. The red labeled K indicates the site of conjugation identified by tandem MS. (**B**) CID fragmentation resulted in a prominent Y-ion series indicating the loss of glucuronic acid and glucose, but no significant peptide backbone fragments. (**C**) ETD fragmentation of the peptide backbone confirmed peptide identity as well as K242 as the site of conjugation. (**D**) Extracted ion chromatogram of doubly charged ions of unconjugated and conjugated peptide 242–249 showing the separation of isobaric conjugate products by C18 LC [overlay of *m/z* = 504.40 (unconjugated peptide, black line), 568.85 (peptide with conjugated linker, blue line) and 928.45 (peptide with conjugated glycan, red line)]. Despite the proximity of the two potential conjugation sites, K242 was more effectively conjugated.

The problem could be avoided by the use of alternative proteases not cleaving at lysine residues (Glu-C, Asp-N). However, these proteases produced peptides with multiple lysine residues. In order to elucidate the conjugation sites it was crucial to acquire sufficient sequence information from the MS/MS experiment. Besides the qualitative data an in-depth conjugation site analysis also required quantitative information on site occupancy. The protease assisted bottom-up approach, however, primarily provided qualitative data since a direct mass spectrometric quantitative comparison between different peptide backbones is impossible. Despite the fact that in some cases the conjugation reactivity of individual lysine residues located on the same peptide could be determined (Fig. 2D), a protease based approach appeared to be unsuitable for any broad quantitative comparison of region/site specific conjugation efficiencies between different conjugate vaccines.

### CRM_197_ neoglycopeptides exhibit unusual CID and ETD fragmentation patterns

Naturally occurring glycopeptides usually exhibit strong and specific oxonium ions when subjected to collision induced dissociation (CID)^28^,^29^. This feature is frequently applied to quickly filter glycopeptide spectra from the bulk of MS/MS data. However, CRM_197_ neoglycopeptides carrying the glyco-epitope constructs used in this study (Table 1) produced a different fragmentation pattern which exhibited an extremely prominent Y-ion series when subjected to CID while showing almost no or very low intensity oxonium ions (see Fig. 2B and Supplementary Fig. S3). Assuming that the majority of protons are associated with the peptide rather than with the glycan moiety, it appears that the linker construct prevents the protons from effectively migrating to the glycan fragments, resulting mostly in the detection of neutral loss fragments. This observed phenomenon appeared to be independent of the length of the carbon spacer. Despite the lack of oxonium ions the observed Y-ions showed a characteristic serial neutral loss of each monosaccharide in the glycan chain. This feature was used to distinguish MS/MS spectra of conjugated peptides from unconjugated ones. CID analyses also enabled confirmation of the glyco-epitope integrity after conjugation to the protein.

Electron transfer dissociation (ETD) of the peptide backbone^30^ enabled the acquisition of sufficient data to confirm peptide identities and map the site(s) of conjugation on the majority of the detected glycopeptides (Fig. 2C). Nevertheless, the ETD MS/MS spectra derived from the CRM_197_ neoglycopeptides showed unusual fragmentation patterns. Z-ions were highly underrepresented, especially when obtained from tryptic glycopeptides. The c-ions series continued at most until the amino acid neighbouring the conjugated lysine, and z-ions containing this residue could generally not be detected.

### Glyco-Epitope screening by nanoLC-ESI-MS/MS

Liquid chromatography enabled separation of isobaric modified peptides carrying the conjugate at different lysine residues. In addition to the glycoconjugates we detected conjugates of adipic acid formed by residual linker reacting with the lysine residues of CRM_197_. This is a common problem since the di-succinimido-adipate (DSA) linker hydrolyses very easily hampering an extensive purification. These adipic acid conjugation artefacts are referred to as "free linker" in this study. Addition of the glycan construct as well as of the free linker resulted in an increased retention time with the glycan-linker construct showing the biggest impact (Fig. 2D).

**Figure 3:**
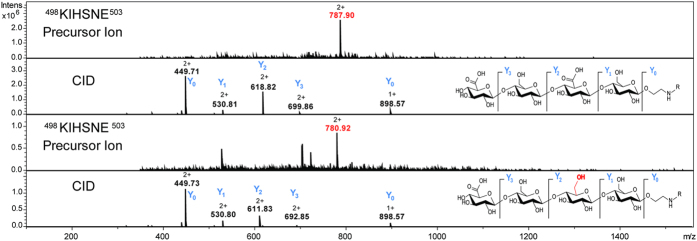
MS spectra of the doubly charged precursor ions *m/z* = 787.90 and *m/z* = 780.92 as well as their respective CID MS/MS spectra. Using CID fragmentation even minor amounts of synthesis byproducts containing glucose instead of a glucuronic acid at the second position could be identified after being conjugated to CRM_197_.

Minor unintended structural features or contaminations might not always be picked up prior to conjugation. The multiple dimensions of nano LC-ESI-MS/MS analysis detected low abundant features such as a peptide carrying a minor side product of the chemical synthesis of ST3. CID fragmentation revealed that one of the two glucuronic acids of the tetrasaccharide was substituted by a glucose resulting in a mass shift of −14 Da from the expected value (Fig. 3). This technique also allows detection and monitoring of any mass modification due to degradation.

Overall, the bottom up approach provided useful data to confirm conjugate integrity, to enable conjugation site mapping and to monitor site-specific conjugation preferences. Nevertheless, issues such as incomplete sequence coverage or the uncontrollable heterogeneity of proteolytic (glyco)peptide products^31^ made it less suited for ensuring batch-to-batch consistency of chemically glycosylated CRM_197_ in a pharmaceutical context. Therefore, an orthogonal LC-MALDI-TOF MS based approach was developed with the intention to overcome these shortcomings and enable fast batch-to-batch comparisons while still providing more detailed data on glycosylation occupancy compared to intact protein analyses.

### Improved CRM_197_-glycoconjugate sequence coverage using chemical cleavage combined with LC-MALDI-TOF-MS

We developed and applied an LC-MALDI-TOF-MS middle-down proteomics approach based on CNBr mediated cleavage to increase sequence coverage and simplify batch-to-batch comparisons while achieving a better general overview on the entire protein sequence detecting any intended and unintended modifications. CNBr mediated protein cleavage results in larger glycopeptides that should compensate or significantly reduce ionisation suppression effects. Glycosylation was shown to reduce ionisation efficiency of tryptic N-glycopeptides, however ionisation efficiency appeared to increase when the peptide to glycan mass ratio was shifted towards the peptides^32^.

**Figure 4:**
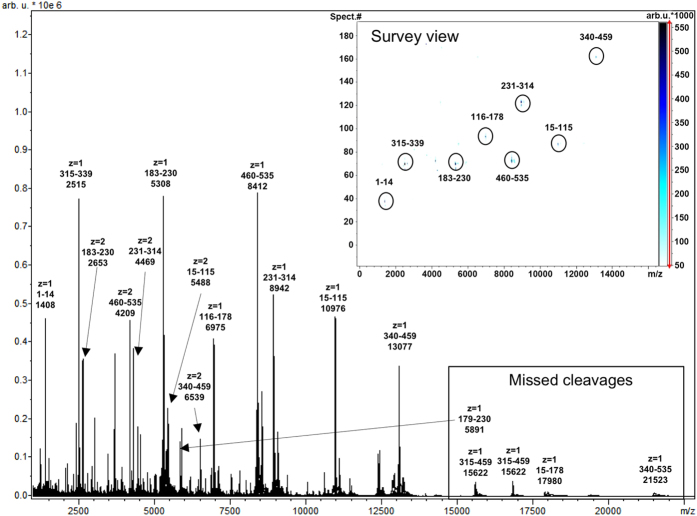
Overlay of all LC-MALDI-MS spectra acquired during the LC run of unconjugated CRM_197_. For each identified peak charge, peptide position and average mass are indicated. The major peaks are all corresponding to the singly charged CNBr peptides without any missed cleavages. All of the eight expected peptides were detected. Doubly charged species were also found for larger peptides as well as low abundant signals corresponding to peptides with missed cleavage sites. The survey view illustrates retention time differences of the various peptides.

CNBr cleavage occurs on the C-terminal side of methionine residues^33^. In the case of CRM_197_ CNBr cleavage results in nine distinct (glyco)peptides for subsequent LC-MALDI-TOF-MS analysis (see Supplementary Table S4). Eight out of nine peptides could be reproducibly detected, corresponding to a sequence coverage of > 99% (Fig. 4). The missing four amino acid peptide (^179^YEYM^182^) exhibited a mass < 600 Da, which is below the optimal operation range of MALDI-TOF-MS. This particular peptide is one of two that do not carry any potential conjugation sites such as lysine residues or the protein's N-terminus, ultimately leaving only seven peptides that needed to be considered in a simplified and comprehensive batch-to-batch comparison.

Due to the large size of the peptides C8- as well as C4-reversed phase LC were tested for (glyco)peptide separation. The C4-chromatography provided better peak shapes and separation reproducibility and was therefore used for the majority of analyses. As expected, the most intense *m/z* signals corresponded to singly charged ions while larger peptides (*m/z* > 5000 Da) were detected as minor signals of doubly charged species as well (Fig 4).

Two batches of ST3 conjugate were analysed in a first run. Depending on their size, the peptides usually eluted during approx. 1–2 minutes. ST3 conjugated glycopeptides were detected by their specific mass shift of + 847.80 Da, corresponding to the average mass of one glycoconjugate unit. A mass shift of + 128.06 Da indicated the presence of free linker.

**Figure 5:**
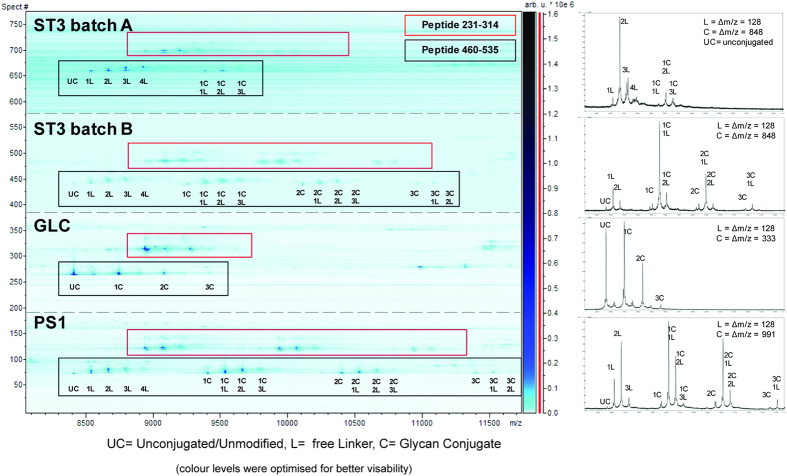
Comparison of conjugation efficiency on peptides 231–314 and 460–535 shown for four samples carrying different glycoepitopes. In the SurveyViewer (retention time vs. *m/z)* of a (C4) LC-MALDI-MS experiment (left) intensities of the detected signals are plotted vs. their retention times and signal intensities in a gel-like representation, providing a quick overview on the number of conjugates present on each CNBr peptide (right). Spectra obtained for peptide 460–535 at a given retention time. Samples conjugated with the DSA-linker appeared in clusters with varying amounts of linker and glycoconjugates. Linker addition induced a shift of + 128 Da and minor shifts in retention time. A single glycoconjugate addition increased the mass by 848 Da (ST3), 333 Da (GLC), or 991 Da (PS1), respectively. Major differences in loading of free linker and glycans were easily detectable by this approach. ST3 batch-to-batch comparison revealed batch A containing up to one glycan conjugated to peptide 231–314 and up to two conjugated to peptide 460–535, whereas the loading was increased in batch B with up to two glycoconjugates attached to peptide 231–314 and up to three to peptide 460–535, respectively. The lower glycan loading in batch A correlated with stronger signals for linker conjugation. GLC was conjugated via a PNP-linker and showed no detectable free linker additions and therefore no clusters as seen for the DSA-conjugated peptides.

The presence of conjugated adipic acid on a CNBr peptide resulted in a retention time shift of around + 30 seconds whereas the presence of a glycoconjugate barely had any effect. In contrast, in the bottom up approach the separation of (glyco)peptides using C18 chromatography, free linker and especially glycoconjugates showed a much larger influence on retention time (Fig.  5). The survey view of the WARP-LC software allows for a simultaneous assay like representation of retention time, *m/z* and intensity. This provided a clear overview of the conjugation status of each CNBr cleaved (glyco)peptide. Increasing amounts of glycoconjugates as well as the addition of several adipic acid units could be easily depicted as characteristic peak patterns (Fig. 5).

### SurveyViewer assisted comparison of batch-to-batch variances

**Figure 6:**
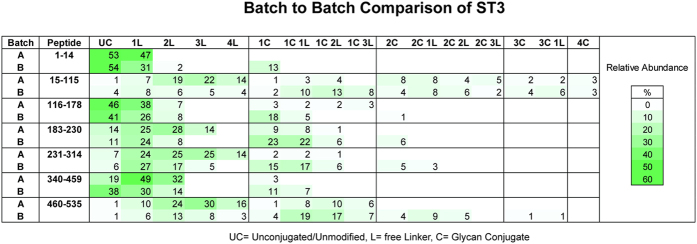
Relative quantitation of the seven lysine containing CNBr peptides derived from two ST3 batches. Each peptide section was quantified individually. The sum of the area under the curve from all detected signals obtained for a given peptide (with and without modifications) was set to 100 %. The percentages presented in the graph are rounded to the full digit. Differences in glycan and free linker loading could be directly compared for each peptide. The results clearly demonstrated that glycan loading was more effective in batch B over the entire protein sequence. Inversely batch A exhibited higher loading of free linker molecules.

Analyses of several batches of conjugates demonstrated that differences in loading and the extent of side reactions could be quickly detected in a protein region specific manner and evaluated using this approach. Major differences in free linker loading and consequently in glycan loading could be observed between the different conjugates (Fig. 5). Batch B showed one additional glycan conjugation in each of the peptides whereas batch A exhibited an increased free linker loading. More detailed information was obtained when relative quantification of the respective (glyco)peptides was applied (Fig. 6). The degree of conjugation with the free linker differed considerably in the two batches. Batch A generally displayed an elevated loading. This increased occupation of lysine residues with free linker molecules resulted in a reduced number of available sites for glycan conjugation, explaining the observed lower glycan loading.

To address the issue of adipic acid conjugation onto the protein and further benchmark the assay a new set of CRM_197_ glycoconjugate was prepared using the *p*-nitrophenol (PNP) activation method^34^ with GLC as a test epitope (Table 1, Fig. 5). Compared to DSA, the PNP-linker is less prone to hydrolysis allowing an extensive purification after activation. The analysis showed that glycan loading was generally lower, however only conjugated glycans and no free linker molecules were detected in both batches. Our findings demonstrate the potential of the assay to reliably detect and differentiate intended and unintended modifications occurring on CRM_197_.

### Evaluation of CNBr cleavage conditions on glycan conjugate integrity

CNBr cleavage requires highly acidic conditions (50% TFA). Even though the sample preparation is performed at low temperature (4 °C) the acidity could potentially affect glycan integrity. Therefore, we evaluated the stability of the glycan epitopes used in this study (Table 1). As exemplified on the ST3, PS1 and GLC-CRM_197_ conjugates no degradation products could be detected (Fig. 5). Our data indicate that most glycan structures appear to tolerate the applied cleavage conditions.

### Conjugation site identification on CNBr glycopeptides using ISD

To determine site-specific attachment information from the conjugated glycopeptides we tested fragmentation by in source dissociation (ISD) using this LC-MALDI-TOF-MS approach. This technique has been frequently used for N- and C-terminal protein sequencing of medium size and larger proteins^35^,^36^,^37^, and in the case of smaller proteins even top-down *de novo* sequencing of the entire protein was achieved^38^.

**Figure 7:**
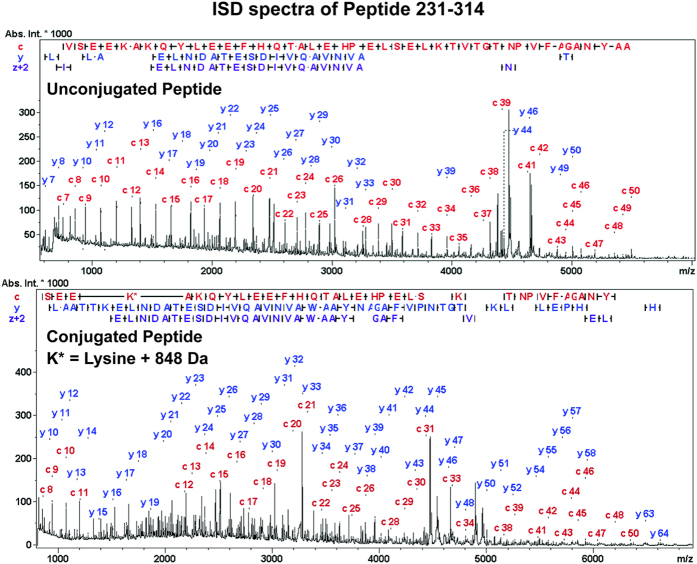
Exemplary MALDI-ISD spectra of CRM_197_ peptide 231–314 acquired from the unconjugated control (top) and an ST3 conjugated batch (bottom) after separation by C8 chromatography. An *m/z* + 848 shift of the c_12-_ion observed between the top and bottom spectra indicated K242 to be the major modified lysine residue in this peptide. The ISD-spectra also provided extensive amino acid sequence data of the peptide.

In order to assign the conjugated glycan to a specific lysine residue, the unconjugated peptide should ideally be LC baseline separated from the different conjugated forms. As mentioned previously in the text just minor retention time differences were observed and the necessary separation required for ISD was not achieved when using the C4 column. In contrast, C8 chromatography resulted in sufficient separation in the case of two singly conjugated (glyco)peptides. ISD analysis of these compounds allowed sequence confirmation of the individual peptides and assignment of the modification to the respective amino acid (Fig. 7).

Despite the LC separation it must be assumed that the selected *m/z* signals still contained a mixture of conjugation site isomers. Therefore, fragment ions specific for less abundant isomers are likely suppressed by the most intense glycopeptide isomer(s), providing information on the site(s) most efficiently modified within a glycopeptide. In peptide 231–314, K242 was identified as the most prominent conjugation site for both, conjugations with the free linker as well as with the glycan. A similar observation was made for K212 on peptide 183–230 (see Supplementary Fig. S5), indicating that these lysine residues are more frequently conjugated. These results also correlated well with the data obtained for K242 within peptide 242–249 after digestion with Glu-C (Fig. 2D) and demonstrate the principal applicability of ISD fragmentation to obtain in-depth attachment site information on chemically conjugated glycopeptides.

### Evaluation of conjugation site occupancy

**Figure 8:**
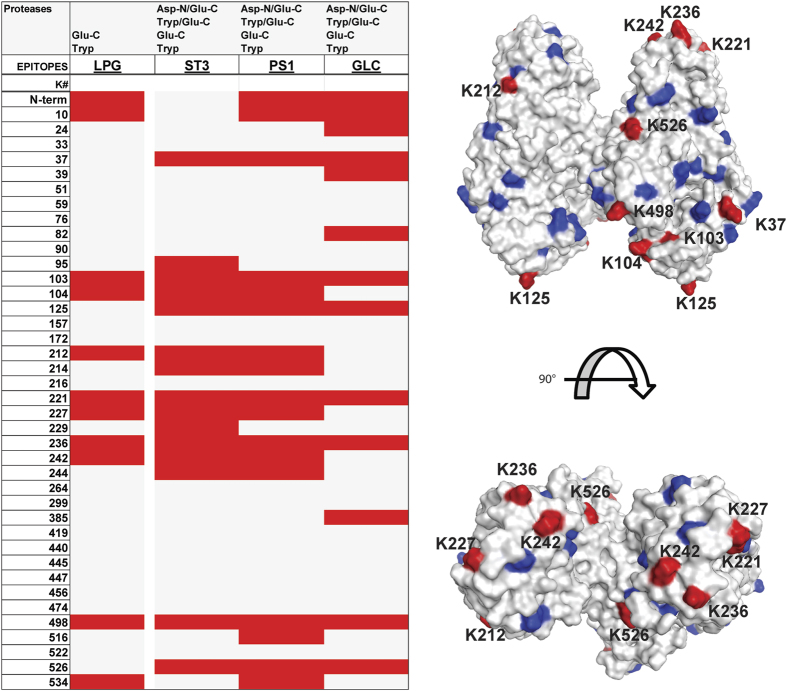
(Left): Heatmap showing the lysine residues detected to be conjugated (red label) after proteolytic digestion with trypsin, Glu-C or combinations of trypsin/Glu-C or Glu-C/Asp-N. Grey labelled lysine residues were not observed as being conjugated. Data obtained for four independent conjugates carrying a variety of glycoepitopes (LPG, ST3, PS1 and GLC as represented in each column) showed that several lysine residues were commonly found conjugated. (Right) 3D crystal structure of CRM_197_ dimer (PDB entry: 4AE0). Lysine residues are labelled blue. Lysine residues that were frequently found conjugated with a glycan in all the samples are labelled in red.

Even though CRM_197_ contains 39 lysine residues and the N-terminal amine that can serve as sites of conjugation, not all of them seem to be equally reactive. Steric accessibility, the individual pKa of the respective lysine residues as well as reaction conditions and conjugate size are likely to influence the conjugation efficacy of individual sites. To evaluate whether particular lysine residues represent favoured sites of conjugation, various CRM_197_ conjugates (ST3, PS1, LPG and GLC, Table 1) were digested either with trypsin or orthogonal proteases and protease combinations. Thereby, a qualitative conjugation site map of all modified primary amines was established based on the presence of conjugation sites in the different samples (Fig. 8).

Three areas within CRM_197_ seem to be preferred for the attachment of glycan epitopes: In addition to lysines K37, K103, K104 and K125, a region including residues K212, K221, K227, K236 and K242 as well as the C-terminal part (in particular K498 and K526) were repeatedly found to be conjugated (Fig. 8). Some peptides such as the peptide ^519^DHTKVNSKLSLFFE^532^ containing K526 could only be detected using certain protease combinations. In terms of attachment sites the results are in good agreement with those from Crotti *et al.*^20^, who evaluated site reactivity based on the presence of a linker molecule without the glycan. *In silico* mapping of the lysine residues on a CRM_197_ structure model^39^ showed that most conjugation sites identified in the course of this work are located on outer edges (Fig. 8).

The conjugation of mono- to pentasaccharides as performed in this study as well as the conjugation of just a linker molecule as described by Crotti and colleagues did not show marked differences in conjugation site selectivity. Based on these data we conclude that smaller variations in conjugation efficiency observed within the different epitopes are in majority due to unavoidable batch-to-batch variations resulting from the chemical glycosylation protocol rather than induced by structural differences of the epitopes.

In contrast, the microenvironment of the conjugation site apparently influenced the conjugation efficiency significantly, as demonstrated in particular by glycopeptide ^242^KAKQYLEE^249^ obtained after Glu-C-digestion. The two lysine residues K242 and K244 are located within an alpha helical part in close proximity to each other but differed significantly in their respective conjugation reactivity. An exclusive steric effect is unlikely to account for the reactivity differences, however variations in the regional pKa of the respective lysine residues might be involved. Studies on helix stabilisation by glutamic acid and lysine residues showed that these amino acids, when spaced three to four amino acids apart, were efficiently stabilizing alpha helices by the formation of salt bridges^40^. Unlike K242, K244 is located in sufficient proximity to three glutamic acid residues (E240, E241, E248, Fig. S6) and could be involved in forming a salt bridge, stabilizing the positive charge of K244 and hence decreasing its nucleophilicity, rendering K244 less reactive.

### Semi-quantitative regio-specific occupancy determination by LC-MALDI-TOF MS

**Figure 9:**
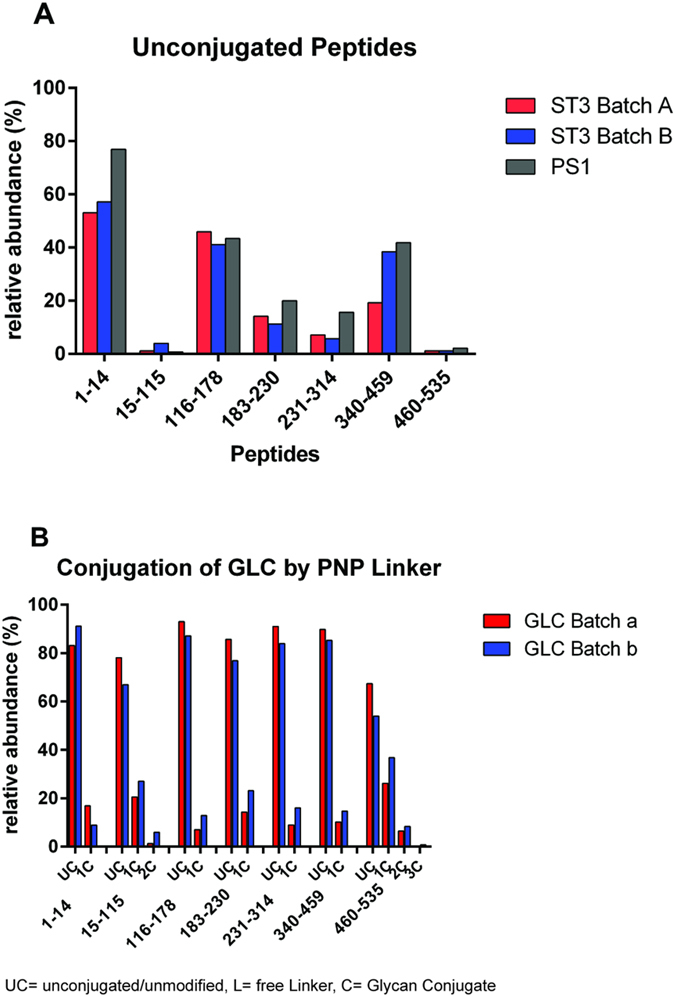
Quantitation results from LC-MALDI-MS measurements. (**A**) Quantitation of unconjugated peptides from of ST3 batch A, ST3 batch B and PS1 (only peptides containing lysine residues were taken into account). Peptides 15–115 and 460–535 show the lowest amounts of unconjugated peptide, consequently carrying larger amounts of glycoconjugates and/or free linker, indicating that conjugation was more efficient at lysine residues present in these peptides. (**B**) Quantitation of two batches of GLC (batch a and batch b) conjugated via the PNP-linker. Only glycan conjugates were found to be present on the peptides. Again, peptides 15–115 and 460–535 show the highest loading.

The signal intensities of the unconjugated peptide and all of its modified forms detected in the LC-MALDI-TOF MS experiments were summed up and the relative amounts for each peptide/glycopeptide group were determined. In the case of ST3 and PS1, peptides 15–115 and 460–535 were most efficiently conjugated, followed by peptides 183–230 and 231–314 (Fig. 9A). Peptides 1–14, 116–178 and 340–469 were found to be least efficiently conjugated. A similar result was obtained for GLC-CRM_197_ conjugates that were produced via the PNP method, where no free linker interfered with the conjugation. Again, peptides 15–115 and 460–535 showed the lowest amount of unconjugated peptide and the highest conjugate loading (Fig. 9B). Apart from peptide 1–15, the LC-MALDI-MS results for the least conjugated region are qualitatively correlating with the LC-ESI-MS data.

The data acquired using various orthogonal approaches in the course of this study allowed us to semi-quantitatively evaluate conjugation efficiency on the individual lysine residues. We concluded that amino acid residues K103, K498, and K526 were among the most reactive ones in the peptides 15–115 and 460–535, since these peptides showed the highest overall conjugation. In the slightly less reactive peptides 183–230 and 231–314 amino acids K212, K221, K242 and K236 were among the frequently conjugated sites.

## Conclusions

Characterisation of glycoconjugate vaccines requires sophisticated and orthogonal approaches. In the course of this study we applied well-established glycoproteomic techniques and developed a novel approach for the in-depth characterisation of the widely used immunogenic carrier protein CRM_197_ that was modified with a variety of defined synthetic oligosaccharide epitopes. Chemical cleavage using CNBr followed by a "middle-down” LC-MALDI-ISD detection strategy provided several advantages towards any protease based assays for comprehensive and in-depth semi-quantitative evaluation of region specific conjugation efficiency for CRM_197_ glycoconjugate vaccine candidates. This technique largely facilitated conjugation product screening by providing virtually complete sequence coverage, good overview on intended as well as unintended modifications and allowed a reproducible, protease independent opportunity for batch-to-batch evaluations. From the 40 possible primary amines (39 lysines + N-terminus) present in CRM_197_, just a few participate in quantitatively relevant conjugation reactions. Steric accessibility, the local amino acid environment and protein secondary structure are likely the most relevant parameters influencing the conjugation reaction under non-denaturing conditions. Our findings can ensure quality control of effective and safe vaccines as well as improving rational vaccine design in the future. It remains to be seen how conjugation variations on these specific sites possibly influence CRM_197_ conjugate vaccines effectiveness, nevertheless the presented assay provides an important step towards functional studies applying CRM_197_ glycoconjugate vaccine candidates carrying defined synthetic glyco-epitopes.

## Material & Methods

### Reagents

Sequencing grade trypsin was purchased from Roche (Mannheim, Germany), Glu-C and Asp-N from Protea Bioscience (Morgantown, WV). Acetonitrile (ACN), formic acid (FA), trifluoroacetic acid (TFA), cyanogen bromide (CNBr), iodoacetamide (IAA), dithiothreitol (DTT), ammonium bicarbonate and triethylamine amine were purchased from Sigma Aldrich (Munich, Germany). Recombinant CRM_197_ was obtained from Pfenex (San Diego, CA). The BCA assay was purchased from Pierce (Rockford, IL), C_18_ ZipTips® and Cetricon® diafilters were purchased from Millipore (Tullagreen, Irland) and hydrophilic lipophilic balanced solid phase extraction cartridges 30 MG (HLB SPE) were obtained from Supelco/Sigma Aldrich (Bellefonte, PA). Generally, the MIAPE and MIRAGE reporting guidelines are followed throughout this work^41^,^42^.

### Glycan epitope synthesis

The glycan epitopes ST3, PS1 and LPG (Table 1) were synthesised from monosaccharide building blocks as described previously^43^,^44^,^45^.

### Synthesis of p-nitrophenol activated ester (GLC-PNP)

The details on the synthesis of GLC-PNP are described in Supplementary Material S7.

### DSA conjugation of synthetic glycans to CRM_197_

Oligosaccharide (3.46 μmol) was dissolved in 100 μL DMSO and added drop wise to a solution of 0.33 mmol of di-succinimido adipate in 100 μL DMSO. Before the addition of glycan, a catalytic amount of triethylamine was added to the linker solution. After 2 hours 0.5 mL 0.1 M phosphate buffer pH 7.4 was added to the reaction mixture and residual unreacted linker was extracted with 14 mL of Chloroform. The extraction procedure was repeated three times and the resultant aqueous layer was centrifuged (300 g, 5 min) to separate traces of chloroform. The resultant aqueous layer was added to 1 mL of protein solution (CRM_197_ 1 mg/mL in 0.1 M phosphate buffer pH 7.4) and the reaction was allowed to continue for 5–6 hours with gentle stirring. The final reaction mixture was purified by ultrafiltration and the final protein concentration was determined by micro BCA assay following the manufacturer’s recommendations.

### *p*-Nitrophenol (PNP) conjugation of synthetic glycans to CRM_197_

PNP half ester (172.4 nmol, 4 μg/μL in DMSO) was added slowly to 17.24 nmol of CRM_197_ in 500 μL of 0.1 M phosphate buffer pH 7.9 and allowed to react for 24 h at room temperature. After the reaction was complete, the protein was dialysed by diafiltration (30 kDa Centricon Diafilters). Conjugation efficiency was monitored by SDS-PAGE and MALDI-TOF MS.

### Proteolytic Digestion

Protein (5 μg) was added to 4 μL of a 4x SDS-PAGE sample buffer (250 Mm Tris-HCl, 40% glycerine, 4% SDS, 0.015% Bromophenol blue containing 50 mM DTT) and denatured at 96 °C for 5 minutes before 0.5 μL of 500 mM IAA were added to achieve a final concentration of 50 mM IAA. The solution was incubated 30 min in the dark before loading the protein onto a 10% SDS-PAGE gel for electrophoretic separation. Proteolytic digestion and peptide extraction were performed as described earlier^28^. Briefly, excised bands were cut into pieces destained in 50% ACN and dried. Proteases (Trypsin, Glu-C, Asp-N) were used in a protease to protein ratio of 1:50 respectively in the single digests or successively in double digests (Tryp/Glu-C; Glu-C/Asp-N). After peptide extraction with 2x 50% ACN and 2x 5% FA the samples were dried in a Speed Vac concentrator and reconstituted in 0.1% FA for further MS analysis.

### Cyanogen bromide cleavage

One crystal of cyanogen bromide (CNBr) was dissolved in 200 μL 50% TFA. 10 μg (30 μg for larger amounts) of protein were dissolved in 190 μL 50% TFA. Subsequently, 10 μL of the CNBr stock solution were added. The solution was topped with N_2_, sealed with Parafilm and incubated for 48 hours at 4 °C in the dark. The reaction was quenched by the addition of 1 mL of water with subsequent removal of the liquid by Speed Vac drying. The sample was reconstituted in 10 μL 0.1% FA and purified by C_18_ ZipTips eluting in 10 μL 80% FA. Larger amounts of initial protein were reconstituted in 50 μL and purified by HLB SPE. The sample was washed three times with 400 μL of 0.1% FA and peptides were eluted using three times 200 μL 80% ACN containing 0.1% FA. The sample was dried and reconstituted in 20 μL of 0.1% TFA for LC-MALDI-TOF-MS analysis.

### LC-ESI-MS/MS

NanoLC-ESI-MS analysis was carried out on an Ultimate 3000 RSLC-nano system (Dionex/Thermo Scientific, Sunnyvale, CA) coupled to an amaZon speed ETD ion trap (Bruker, Bremen, Germany). In each run peptides corresponding to 0.15 μg of a CRM_197_ digest were injected. The peptides were concentrated on a C18 precolumn (Acclaim PepMap100™, Thermo, 100 μm × 20 mm, 5 μm particle size) and separated by reversed phase chromatography on a C18 analytical column (Acclaim PepMAp™, Thermo, 75 μm × 15 cm, particle size 3 μm). The samples were loaded in 99% buffer A (0.1% FA) for 6 min on the precolumn at a flow rate of 6 μL/min before the captured peptides were subjected to nanoLC at a flowrate of 350 nL/min using a gradient of increasing ACN as follows: buffer B (ACN containing 0.1% FA) from 1% to 20% (7–57 min) followed by a further increase to 50% (57–75 min) and a steep increase to 90% (75–86 min) before returning to the starting conditions. The mass spectrometer was set-up to perform CID as well as ETD fragmentation on the three most intense signals in every MS scan. An *m/z* range from 380–1800 Da was used for data dependent precursor scanning. The MS data was recorded using the instrument's "enhanced resolution mode". MS/MS data was acquired in "ultra-mode" over an *m/z* range from 100–2000. Details on the MS CID and ETD settings can be found as Supplementary Table S1.

### LC-MALDI-TOF-MS analysis

LC-MALDI-TOF-MS experiments were performed on an ultrafleXtreme MALDI-TOF/TOF (Bruker, Bremen, Germany). Peptides were separated via an Agilent 1200 by C8 (Zorbax 300SB-C8, 3.5 μm, 100 × 0.3 mm from Agilent) and C4 (Phenomenex, Jupiter C4 300A, 150 × 0.3 mm) capillary reversed phase separation. Typically, 4–8 μg of samples were injected. Buffer A was 0.1% TFA. Buffer B was ACN with 0.1% TFA. The 40 min gradient was as followed: Buffer B: 5–20% in 5 min and 20–55% over 30 min. Fractions of 3 μL were spotted every 15 seconds by a PROTEINEER fc II on a MTP BigAnchor 384 MALDI-target (both Bruker, Bremen, Germany). Super dihydroxybenzoic acid (sDHB) was used as matrix. Aliquots of 0.5 μL of a 50 mg/mL matrix solution in 50% ACN/water/0.1% TFA were spotted to each dried fraction.

The fractions were then analysed in an automated way by WARP-LC 1.3 and Compass 1.4 in positive linear ion mode and spectra were displayed according to their retention time in the SurveyViewer of the software. In-source decay (ISD) spectra were then manually acquired on selected fractions using a positive reflector method optimised for ISD. Typically, 5000–10000 laser shots were accumulated for each ISD spectrum. External calibration of ISD spectra was performed with ISD fragments of bovine ubiquitin spotted on the calibration chips of the MTP 384 BigAnchor target.

### MS Data Processing

LC-MS data was analysed with Compass 4.1 analysis software (Bruker, Bremen, Germany). Compound spectra were created with a retention time window of 0.5 min and an intensity threshold of 100000. EIC for neutral loss of respective monosaccharide glycan masses were screened for conjugated peptides as well as all compound spectra were checked manually for presence of conjugated peptides. Protein coverage was evaluated using ProteinScape 3.1 (Bruker, Bremen, Germany) and the MS data was searched against a custom-made database using Mascot server 2.3 including all available entries for CRM and diphtheria toxin. The cleavage sites were defined as non-specific since some unspecific cleavage products were identified.

LC-MS data was analysed and the area under the curve (AUC) was quantified on SurveyViewer (Bruker). For quantitation AUC of the EICs of the respective peptide/glycopeptide signals was integrated. For each peptide the sum of all AUC of the modified forms and the non-modified peptides was set as 100%. The ISD spectra were processed in Compass 4.1 using SNAP as peak picking algorithm for monoisotopic peak annotation and further analysed in BioTools 3.2 SR4 (Bruker). Peptide sequences were modified with the conjugates and matched with the ISD spectra to determine the modification sites. Each annotation was further validated manually. The mapping of the reactive lysine residues in the 3D model was performed using Molecular Operating Environment (MOE), 2014.09 (Chemical Computing Group Inc., 1010 Sherbooke St. West, Montreal, QC, Canada).

## Additional Information

**How to cite this article**: Möginger, U. *et al.* Cross Reactive Material 197 glycoconjugate vaccines contain privileged conjugation sites. *Sci. Rep.* **6**, 20488; doi: 10.1038/srep20488 (2016).

## Supplementary Material

Supplementary Information

## Figures and Tables

**Figure 1:**
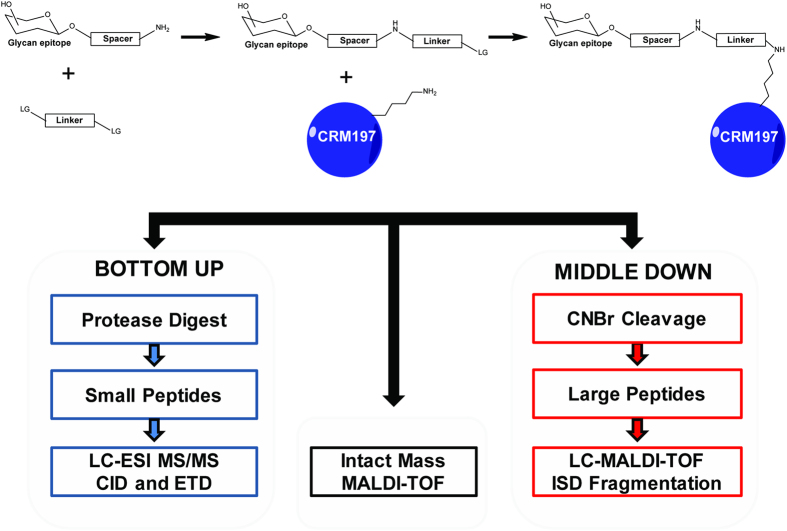
Schematic overview of the three orthogonal approaches employed for the in-depth characterisation of CRM_197_ conjugated with defined synthetic minimal glycoepitope vaccine candidates. Step 1: The glycan part is synthesised with a spacer carrying an amine group. Step 2: addition of the linker molecule with two leaving groups (LG). Step 3: addition of the glycan linker construct to the carrier protein and subsequent conjugation to a primary amine (e.g. lysine residue).

**Table 1:** Overview on linker, spacer and glycan epitope constructs used in this study.

LINKER AND SPACER MOLECULES				
Structure	Abbreviation	Composition	Description	
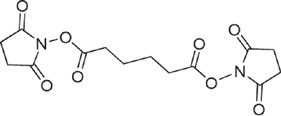	DSA	C_14_H_16_N_2_O_8_	di-succinimido-adipate linker *Leaving group: succinimide*	
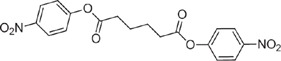	PNP	C_18_H_16_N_2_O_8_	Adipic acid *p*-nitro-phenol linker *Leaving group: para-nitrophenol*	
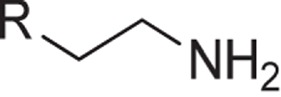	C2	C_2_H_6_N	2 carbon unit spacer	
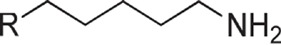	C5	C_5_H_12_N	5 carbon unit spacer	
Linker side product (depicted as present after conjugation)				
Structure	Abbreviation	Composition	Δ*m/z*	Description
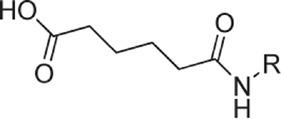	L	C_6_H_10_O_3_	128.06	Adipic acid/free linker #
CONJUGATES (depicted as present after conjugation)				
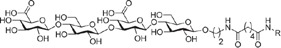	ST3	(GlcA)2(Glc)2-C2- DSA	847.80	Epitope from *Streptococcus pneumoniae* serotype 3
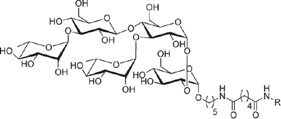	PS1	(Rha)2(Glc)3-C5-DSA	991.42	Epitope from *Clostridium difficile*
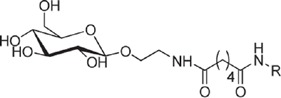	GLC	Glc- C2-PNP	333.14	Glucose test glycan
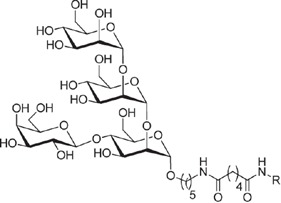	LPG	(Man)3(Gal)-C5-DSA	861.36	Lipophosphoglycan capping oligosaccharide *Leishmania infantum*

Δ*m/z*: monoisotopic mass addition upon conjugation to a primary amine. Glc = glucose, Rha = rhamnose, GlcA = glucuronic acid, Hep = heptose, Man = mannose, Gal = galactose; # Conjugation of linker molecule and subsequent hydrolysis of the leaving groups results in the same product for DSA linker and PNP linker.

## References

[b1] Bazaka K., Crawford R. J., Nazarenko E. L. & Ivanova E. P. Bacterial Extracellular Polysaccharides. In: Bacterial Adhesion (eds Linke D., Goldman A. ). 715, 213–226. Springer: Netherlands, (2011). 10.1007/978-94-007-0940-9_13 21557066

[b2] Stein K. E. Thymus-independent and thymus-dependent responses to polysaccharide antigens. J of infec Dis 165 Suppl 1, S49–52 (1992). 1588177 10.1093/infdis/165-supplement_1-s49

[b3] Avery O. T. & Goebel W. F. Chemo-immunological studies on conjugated carbohydrate-proteins: II. Immunological specificity of synthetic sugar-protein antigens. J Exp Med 50, 533 (1929). 19869645 10.1084/jem.50.4.533 PMC2131643

[b4] Peltola H., Kayhty H., Sivonen A. & Makela H. Haemophilus influenzae type b capsular polysaccharide vaccine in children: a double-blind field study of 100,000 vaccinees 3 months to 5 years of age in Finland. Pediatrics 60, 730–737 (1977). 335348

[b5] Pon R. A. Exploiting the Bacterial Surface: The Successfull Application of Glycoconjugate Vaccines In: Bacterial glycomics: current research, technology and applications (eds Reid C. W., Reid A. N., Twine S. M. ), 243–262. Horizon Scientific Press (2012).

[b6] Giannini G., Rappuoli R. & Ratti G. The amino-acid sequence of two non-toxic mutants of diphtheria toxin: CRM45 and CRM_197_. Nucleic Acids Res 12, 4063–4069 (1984). 6427753 10.1093/nar/12.10.4063 PMC318816

[b7] Shinefield H. R. Overview of the development and current use of CRM_197_ conjugate vaccines for pediatric use. Vaccine 28, 4335–4339 (2010). 20452430 10.1016/j.vaccine.2010.04.072

[b8] Jin S. D. *et al.* Optimization of capsular polysaccharide production by Streptococcus pneumoniae type 3. J Microbiol Biotechnol 19, 1374–1378 (2009). 19996690 10.4014/jmb.0903.3027

[b9] Bertolo L. *et al.* The design of a capsule polysaccharide conjugate vaccine against Campylobacter jejuni serotype HS15. Carbohydr Res 366, 45–49 (2013). 23261782 10.1016/j.carres.2012.11.017

[b10] Pujar N. S. *et al.* Base hydrolysis of phosphodiester bonds in pneumococcal polysaccharides. Biopolymers 75, 71–84 (2004). 15307199 10.1002/bip.20087

[b11] Frasch C. E. Preparation of bacterial polysaccharide-protein conjugates: analytical and manufacturing challenges. Vaccine 27, 6468–6470 (2009). 19555714 10.1016/j.vaccine.2009.06.013

[b12] Anish C., Schumann B., Pereira Claney L. & Seeberger Peter H. Chemical Biology Approaches to Designing Defined Carbohydrate Vaccines. Chem Biol 21, 38–50 (2014). 24439205 10.1016/j.chembiol.2014.01.002

[b13] Verez-Bencomo V. A Synthetic Conjugate Polysaccharide Vaccine Against Haemophilus influenzae Type b. Science 305, 522–525 (2004). 15273395 10.1126/science.1095209

[b14] Seeberger P. H. & Werz D. B. Synthesis and medical applications of oligosaccharides. Nature 446, 1046–1051 (2007). 17460666 10.1038/nature05819

[b15] Costantino P., Rappuoli R. & Berti F. The design of semi-synthetic and synthetic glycoconjugate vaccines. Expert Opin Drug Discov 6, 1045–1066 (2011). 22646863 10.1517/17460441.2011.609554

[b16] Chu C. Y. *et al.* Preparation, characterization, and immunogenicity of conjugates composed of the O-specific polysaccharide of Shigella dysenteriae type 1 (Shiga's bacillus) bound to tetanus toxoid. Infect Immun 59, 4450–4458 (1991). 1937803 10.1128/iai.59.12.4450-4458.1991 PMC259062

[b17] Broker M., Dull P. M., Rappuoli R. & Costantino P. Chemistry of a new investigational quadrivalent meningococcal conjugate vaccine that is immunogenic at all ages. Vaccine 27, 5574–5580 (2009). 19619500 10.1016/j.vaccine.2009.07.036

[b18] Peeters J. M., Hazendonk T. G., Beuvery E. C. & Tesser G. I. Comparison of four bifunctional reagents for coupling peptides to proteins and the effect of the three moieties on the immunogenicity of the conjugates. J Immunol Methods 120, 133–143 (1989). 2499636 10.1016/0022-1759(89)90298-6

[b19] Buskas T., Li Y. & Boons G. J. The immunogenicity of the tumor-associated antigen Lewis(y) may be suppressed by a bifunctional cross-linker required for coupling to a carrier protein. Chemistry 10, 3517–3524 (2004). 15252797 10.1002/chem.200400074

[b20] Crotti S. *et al.* Defined conjugation of glycans to the lysines of CRM_197_ guided by their reactivity mapping. ChemBioChem 15, 836–843 (2014). 24616190 10.1002/cbic.201300785

[b21] Li Q., Rodriguez L. G., Farnsworth D. F. & Gildersleeve J. C. Effects of hapten density on the induced antibody repertoire. ChemBioChem 11, 1686–1691 (2010). 20602400 10.1002/cbic.201000235 PMC3462448

[b22] Desaymard C. & Howard J. G. Role of epitope density in the induction of immunity and tolerance with thymus-independent antigens. II. Studies with 2,4-dinitrophenyl conjugates *in vivo*. Eur J of Immunol 5, 541–545 (1975). 1086248 10.1002/eji.1830050807

[b23] Jones C. The Regulatory Framework for Glycoconjugate Vaccines. In: Carbohydrate-Based Vaccines (eds Roy R. ). 989, 21–35. American Chemical Society (2008).

[b24] Jones C. Vaccines based on the cell surface carbohydrates of pathogenic bacteria. An Acad Bras Cienc 77, 293–324 (2005). 15895165 10.1590/s0001-37652005000200009

[b25] Jones C. & Currie F. Control of components of bacterial polysaccharide vaccines by physical methods. Biologicals 19, 41–47 (1991). 1904745 10.1016/1045-1056(91)90023-d

[b26] Monteiro M. A. *et al.* Capsule polysaccharide conjugate vaccine against diarrheal disease caused by Campylobacter jejuni. Infect Immun 77, 1128–1136 (2009). 19114545 10.1128/IAI.01056-08 PMC2643618

[b27] Allen G. Chapter 3 Specific cleavage of the protein. In: Laboratory Techniques in Biochemistry and Molecular Biology (eds Allen G. ). 9, 43–71. Elsevier (1981).

[b28] Kolarich D., Jensen P. H., Altmann F. & Packer N. H. Determination of site-specific glycan heterogeneity on glycoproteins. Nat Protoc 7, 1285–1298 (2012). 22678432 10.1038/nprot.2012.062

[b29] Wuhrer M., Catalina M. I., Deelder A. M. & Hokke C. H. Glycoproteomics based on tandem mass spectrometry of glycopeptides. J Chromatogr B Analyt Technol Biomed Life Sci 849, 115–128 (2007). 10.1016/j.jchromb.2006.09.041 17049937

[b30] Alley W. R. Jr., Mechref Y. & Novotny M. V. Characterization of glycopeptides by combining collision-induced dissociation and electron-transfer dissociation mass spectrometry data. Rapid Commun Mass Spectrom : RCM 23, 161–170 (2009). 19065542 10.1002/rcm.3850

[b31] Leymarie N. *et al.* Interlaboratory Study on Differential Analysis of Protein Glycosylation by Mass Spectrometry: the ABRF Glycoprotein Research Multi-Institutional Study 2012. Mol Cell Proteomics, (2013). 10.1074/mcp.M113.030643 PMC3790302 23764502

[b32] Stavenhagen K. *et al.* Quantitative mapping of glycoprotein micro-heterogeneity and macro-heterogeneity: an evaluation of mass spectrometry signal strengths using synthetic peptides and glycopeptides. J Mass Spectrom : JMS 48, 627–639 (2013). 23722953 10.1002/jms.3210

[b33] Gross E. & Witkop B. Selective Cleavage of the Methionyl Peptide Bonds in Ribonuclease with Cyanogen Bromide1. J Am Chem Soc 83, 1510–1511 (1961).

[b34] Wu X., Ling C. C. & Bundle D. R. A new homobifunctional p-nitro phenyl ester coupling reagent for the preparation of neoglycoproteins. Org Lett 6, 4407–4410 (2004). 15548037 10.1021/ol048614m

[b35] Suckau D. & Resemann A. MALDI top-down sequencing: calling N-and C-terminal protein sequences with high confidence and speed. J Biomol Tech 20, 258 (2009). 19949698 PMC2777352

[b36] Hardouin J. Protein sequence information by matrix-assisted laser desorption/ionization in-source decay mass spectrometry. Mass Spectrom Rev 26, 672–682 (2007). 17492750 10.1002/mas.20142

[b37] Suckau D. & Resemann A. T3-sequencing: targeted characterization of the N-and C-termini of undigested proteins by mass spectrometry. Anal Chem 75, 5817–5824 (2003). 14588022 10.1021/ac034362b

[b38] Resemann A. *et al.* Top-Down *de Novo* Protein Sequencing of a 13.6 kDa Camelid Single Heavy Chain Antibody by Matrix-Assisted Laser Desorption Ionization-Time-of-Flight/Time-of-Flight Mass Spectrometry. Anal Chem 82, 3283–3292 (2010). 20329751 10.1021/ac1000515

[b39] Malito E. *et al.* Structural basis for lack of toxicity of the diphtheria toxin mutant CRM_197_. Proc Natl Acad Sci USA 109, 5229–5234 (2012). 22431623 10.1073/pnas.1201964109 PMC3325714

[b40] Marqusee S. & Baldwin R. L. Helix stabilization by Glu-...Lys+ salt bridges in short peptides of de novo design. Proc Natl Acad Sci USA 84, 8898–8902 (1987). 3122208 10.1073/pnas.84.24.8898 PMC299658

[b41] Taylor C. F. *et al.* The minimum information about a proteomics experiment (MIAPE). Nat Biotechnol 25, 887–893 (2007). 17687369 10.1038/nbt1329

[b42] Kolarich D. *et al.* The minimum information required for a glycomics experiment (MIRAGE) project: improving the standards for reporting mass-spectrometry-based glycoanalytic data. Mol Cell Proteomics 12, 991–995 (2013). 23378518 10.1074/mcp.O112.026492 PMC3617344

[b43] Martin C. E., Weishaupt M. W. & Seeberger P. H. Progress toward developing a carbohydrate-conjugate vaccine against Clostridium difficile ribotype 027: synthesis of the cell-surface polysaccharide PS-I repeating unit. Chem Commun (Camb) 47, 10260–10262 (2011). 21998885 10.1039/c1cc13614c

[b44] Lefeber D. J., Kamerling J. P. & Vliegenthart J. F. Synthesis of Streptococcus pneumoniae type 3 neoglycoproteins varying in oligosaccharide chain length, loading and carrier protein. Chemistry 7, 4411–4421 (2001). 11695675 10.1002/1521-3765(20011015)7:20<4411::aid-chem4411>3.0.co;2-t

[b45] Liu X. *et al.* Enhancement of the immunogenicity of synthetic carbohydrates by conjugation to virosomes: a leishmaniasis vaccine candidate. ACS Chem Biol 1, 161–164 (2006). 17163663 10.1021/cb600086b

