# Supplementary material for: Cross Reactive Material 197 glycoconjugate vaccines contain privileged conjugation sites
Source: Sci Rep. 2016 Feb 4;6:20488. doi: 10.1038/srep20488 (PMC4740906; doi:10.1038/srep20488)
Supplement: Supplementary Information [file srep20488-s1.pdf]

Supplementary Material for manuscript:

"Cross Reactive Material 197 glycoconjugate vaccines contain privileged conjugation sites"

Authors

Uwe Möginger[1, 2], Anja Resemann[3], Christopher E. Martin[1, 2], Sharavathi Parameswarappa[1], Subramanian Govindan[1§], Eike-Christian Wamhoff[1, 2], Felix Broecker[1, 2], Detlef Suckau[3], Claney Lebev Pereira[1], Chakkumkal Anish[1♦], Peter H. Seeberger[1, 2], Daniel Kolarich[1*]

Affiliations

[1] Department of Biomolecular Systems, Max Planck Institute of Colloids and Interfaces, 14424 Potsdam, Germany

[2] Institute of Chemistry and Biochemistry, Freie Universität Berlin, Germany

[3] Bruker, Bremen, Germany

§ Current address addresses: a) Department of chemistry, Indian Institute of Technology Tirupati, Tirupati-Renigunta Road, Tirupati, Andhra Pradesh, India 517506 and b) Department of Civil Engineering, Indian Institute of Technology Madras, Chennai, Tamil Nadu, India 600036

♦Current address: Bacterial Vaccine Discovery Team, Janssen Pharmaceuticals of Johnson & Johnson, Bioscience Park Leiden, Zernikedreef 9, 2333 CK Leiden, Netherlands

Supplementary Table S1: Ion trap settings applied in this study, following the MIRAGE guidelines (www.beilstein-mirage.org)

**MS Settings - General**	
ESI probe	CaptiveSpray™
Capillary voltage	1.3 kV
SPS	*m/z* 900
Compound stability	100%
Trap Drive Level	100%
Spectra averaging	5
Dry gas temperature	150°C
Dry Gas flow	3 L/min
Maximum accumulation time	200 ms
Ion mode	positive
**MS-Scan**	
MS Scan mode	Enhanced scan
ICC target	200000
Mass detection range	*m/z* 350-1800
**MS2**	
MS scan mode	ultrascan
SPS MS(n)	automatic
MS(n) spectra averages	5
MS(n) ICC target	40000
Preferred charge state	≥Doubly,
Active exclusion	Off
Mass detection range	*m/z* 100-2000
Isolation width	3 Da
Exclude singly charged ions	on
No. of precursor ions	3
SmartFrag	Enhanced
SmartFrag Start amplitude	30%
SmartFrag End amplitude	120%
Fragmentation width	5 *m/z*

27 ProteinScape report on Proteomic Data obtained after LC-ESI MS/MS of proteolytically digested CRM_{197}.

Project Info

Name: CRM

Sample Info & Protocols :

Name: CRMstd: Tryptic Digest

**Protein 1:**	toxin CRM197		
**Accession:**	gi|224021|prf||1007216A	**Score:**	3039.51
**Database:**	allCRM_Kolarich	**MW [kDa]:**	58.40
**Seq. Coverage [%]:**	66.70 %	**pI:**	5.83
		**No. of Peptides:**	64
**Modification(s):**	Carbamidomethyl, Oxidation, Deamidated		

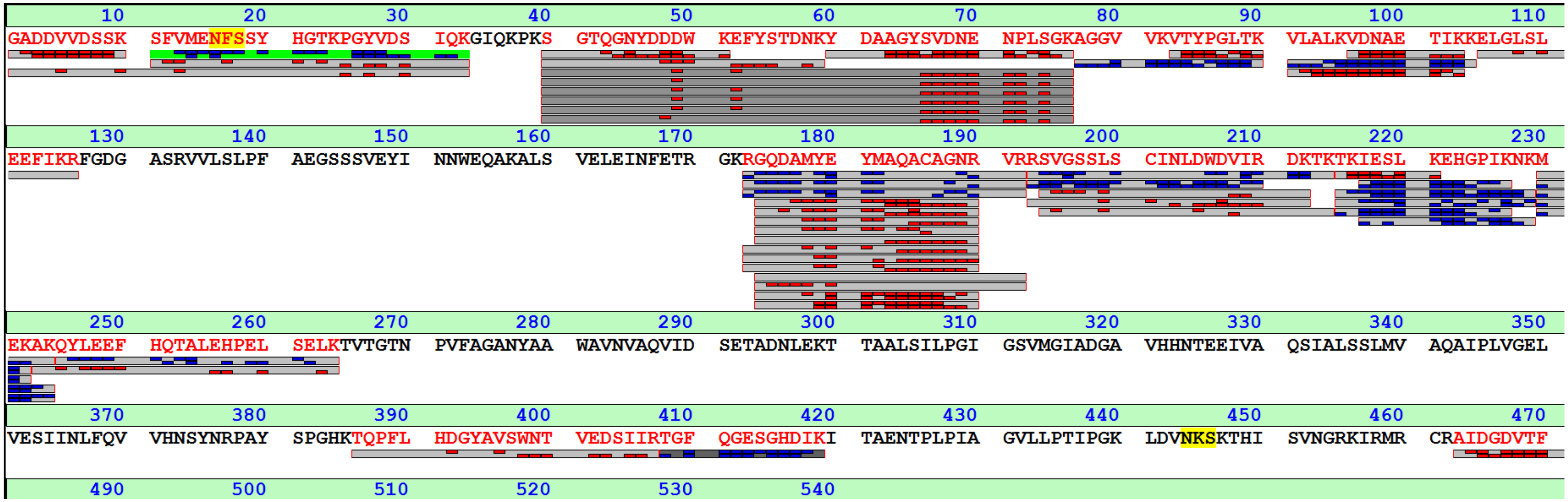

Cmpd.	No. of Cmpds.	*m/z* meas.	Δ *m/z* [ppm]	z	Rt [min]	Score	P	Range	Sequence	Modification
38	5	496.8000	140.55	2	15.2	87.8	0	1-10	-.GADDVVDSSK.S	
390	1	899.4000	-25.34	4	38.4	29.9	1	1-33	-.GADDVVDSSKSFVMENFSSYHGT KPGYVDSIQK.G	
290	2	874.5000	95.83	3	33.3	51.4	0	11-33	K.SFVMENFSSYHGTKPGYVDSIQK .G	
232	1	660.1000	56.51	4	29.7	70.9	0	11-33	K.SFVMENFSSYHGTKPGYVDSIQK .G	Oxidation: 4
95	1	693.3000	23.30	2	20.7	45.8	0	40-51	K.SGTQGNYDDDWK.E	
264	1	790.7000	44.75	3	31.5	30.5	1	40-59	K.SGTQGNYDDDWKEFYSTDNK.Y	
364	1	1039.0000	57.86	4	37.2	26.7	2	40-76	K.SGTQGNYDDDWKEFYSTDNKYDA AGYSVDNENPLSGK.A	Deamidated: 6, 19
364	1	1039.0000	57.86	4	37.2	26.7	2	40-76	K.SGTQGNYDDDWKEFYSTDNKYDA AGYSVDNENPLSGK.A	Deamidated: 4, 19
364	1	1039.0000	57.86	4	37.2	26.7	2	40-76	K.SGTQGNYDDDWKEFYSTDNKYDA AGYSVDNENPLSGK.A	Deamidated: 4, 6
364	1	1039.0000	294.71	4	37.2	23.9	2	40-76	K.SGTQGNYDDDWKEFYSTDNKYDA AGYSVDNENPLSGK.A	Deamidated: 19
364	1	1039.0000	294.71	4	37.2	23.9	2	40-76	K.SGTQGNYDDDWKEFYSTDNKYDA AGYSVDNENPLSGK.A	Deamidated: 6
364	1	1039.0000	294.71	4	37.2	23.9	2	40-76	K.SGTQGNYDDDWKEFYSTDNKYDA AGYSVDNENPLSGK.A	Deamidated: 4
230	2	900.4000	-8.62	2	29.6	95.8	0	60-76	K.YDAAGYSVDNENPLSGK.A	
209	6	464.0000	126.04	3	28.3	82.6	1	77-90	K.AGGVVKVTYPGLTK.V	
135	4	439.8000	107.48	2	23.6	47.4	0	83-90	K.VTYPGLTK.V	
220	2	707.5000	114.19	2	29.2	78.9	1	91-103	K.VLALKVDNAETIK.K	
173	2	514.7000	103.20	3	26.0	70.3	2	91-104	K.VLALKVDNAETIKK.E	
23	2	445.3000	146.20	2	14.0	56.6	0	96-103	K.VDNAETIK.K	
482	3	634.6000	30.37	4	43.9	33.9	1	105-126	K.ELGLSLTEPLMEQVGTEEFIKR. F	Oxidation: 11
122	4	703.3000	5.30	3	22.5	55.4	1	173-190	K.RGQDAMYEYMAQACAGNR.V	Carbamidomethyl: 14; Oxidation: 10
138	4	703.3000	5.30	3	23.8	58.4	1	173-190	K.RGQDAMYEYMAQACAGNR.V	Carbamidomethyl: 14; Oxidation: 6
86	2	708.7000	101.74	3	19.7	55.9	1	173-190	K.RGQDAMYEYMAQACAGNR.V	Carbamidomethyl: 14; Oxidation: 6, 10
90	1	595.8000	65.24	4	20.0	40.8	2	173-192	K.RGQDAMYEYMAQACAGNRVR.R	Carbamidomethyl: 14; Oxidation: 6, 10; Deamidated: 17
90	1	595.8000	65.24	4	20.0	31.5	2	173-192	K.RGQDAMYEYMAQACAGNRVR.R	Carbamidomethyl: 14; Oxidation: 6, 10; Deamidated: 12
90	2	595.8000	478.36	4	20.0	25.9	2	173-192	K.RGQDAMYEYMAQACAGNRVR.R	Carbamidomethyl: 14;

										Oxidation: 6, 10
129	1	657.0000	118.42	3	23.2	30.4	0	174-190	R.GQDAMYEYMAQACAGNR.V	Carbamidomethyl: 13; Oxidation: 5, 9; Deamidated: 16
170	4	651.3000	57.48	3	25.8	68.8	0	174-190	R.GQDAMYEYMAQACAGNR.V	Carbamidomethyl: 13; Oxidation: 5
149	4	651.3000	57.48	3	24.5	47.5	0	174-190	R.GQDAMYEYMAQACAGNR.V	Carbamidomethyl: 13; Oxidation: 9
274	1	968.4000	7.48	2	32.2	71.5	0	174-190	R.GQDAMYEYMAQACAGNR.V	Carbamidomethyl: 13
97	2	984.4000	12.53	2	20.8	67.6	0	174-190	R.GQDAMYEYMAQACAGNR.V	Carbamidomethyl: 13; Oxidation: 5, 9
129	1	657.0000	118.42	3	23.2	23.6	0	174-190	R.GQDAMYEYMAQACAGNR.V	Carbamidomethyl: 13; Oxidation: 5, 9; Deamidated: 2
129	1	657.0000	118.42	3	23.2	22.7	0	174-190	R.GQDAMYEYMAQACAGNR.V	Carbamidomethyl: 13; Oxidation: 5, 9; Deamidated: 11
109	1	742.0000	470.96	3	21.5	76.7	1	174-192	R.GQDAMYEYMAQACAGNRVR.R	Carbamidomethyl: 13; Oxidation: 5, 9
109	1	742.0000	28.68	3	21.5	62.9	1	174-192	R.GQDAMYEYMAQACAGNRVR.R	Carbamidomethyl: 13; Oxidation: 5, 9; Deamidated: 2
463	3	693.1000	113.49	3	43.1	104.8	1	193-210	R.RSVGSSLSCINLDWDVIR.D	Carbamidomethyl: 9
422	1	774.1000	49.11	3	40.2	29.0	2	193-212	R.RSVGSSLSCINLDWDVIRDK.T	Carbamidomethyl: 9
384	2	638.1000	25.12	4	38.1	62.7	3	193-214	R.RSVGSSLSCINLDWDVIRDKTK.T	Carbamidomethyl: 9
464	1	722.1000	99.33	3	43.1	32.0	1	194-212	R.SVGSSLSCINLDWDVIRDK.T	Carbamidomethyl: 8
433	2	798.5000	113.77	3	40.8	42.5	2	194-214	R.SVGSSLSCINLDWDVIRDKTK.T	Carbamidomethyl: 8
36	2	409.8000	115.35	2	15.1	38.5	1	215-221	K.TKIESLK.E	
74	2	494.0000	89.41	3	18.5	61.1	2	215-227	K.TKIESLKEHGPIK.N	
44	3	431.3000	108.59	4	16.1	73.6	3	215-229	K.TKIESLKEHGPIKNK.M	
58	1	431.5000	1.92	4	17.3	27.7	3	215-229	K.TKIESLKEHGPIKNK.M	Deamidated: 14
72	2	417.6000	59.98	3	18.4	63.0	1	217-227	K.IESLKEHGPIK.N	
45	2	498.3000	24.91	3	16.2	38.9	2	217-229	K.IESLKEHGPIKNK.M	
11	5	494.6000	71.87	3	12.4	87.1	1	230-242	K.MSESPNKTVSEEK.A	Oxidation: 1
28	2	489.2000	-67.08	3	14.3	57.1	1	230-242	K.MSESPNKTVSEEK.A	
4	2	561.0000	103.73	3	11.8	68.5	2	230-244	K.MSESPNKTVSEEKAK.Q	Oxidation: 1
14	1	421.2000	-9.93	4	12.8	28.1	2	230-244	K.MSESPNKTVSEEKAK.Q	Oxidation: 1; Deamidated: 6
19	4	555.7000	161.67	3	13.5	55.7	2	230-244	K.MSESPNKTVSEEKAK.Q	
305	1	880.8000	20.83	3	34.0	77.4	1	243-264	K.AKQYLEEFHQTALEHPELSELK.T	
346	1	611.1000	73.58	4	36.3	38.9	0	245-264	K.QYLEEFHQTALEHPELSELK.T	
442	2	850.1000	15.20	3	41.4	45.9	0	386-407	K.TQPFLHDGYAVSWNTVEDSIIR.T	
57	4	425.9000	69.69	3	17.1	73.6	0	408-419	R.TGFQGESGHDIK.I	
152	5	689.9000	82.89	2	24.7	67.0	0	463-474	R.AIDGDVTFCRPK.S	Carbamidomethyl: 9
254	2	692.4000	61.86	3	31.0	27.6	0	475-493	K.SPVYVGNGVHANLHVAFHR.S	Deamidated: 12

**254**	**3**	**692.4000**	**61.86**	**3**	**31.0**	**59.9**	**0**	**475-493**	**K.SPVYVGNGVHANLHVAFHR.S**	**Deamidated: 7**
**225**	**2**	**692.1000**	**102.35**	**3**	**29.4**	**45.8**	**0**	**475-493**	**K.SPVYVGNGVHANLHVAFHR.S**	
**227**	**1**	**1233.6000**	**-2.73**	**2**	**29.4**	**26.4**	**1**	**494-516**	**R.SSSEKIHSNEISSDSIGVLGYQK.T**	**Deamidated: 9**
**213**	**3**	**617.1000**	**65.93**	**4**	**28.7**	**97.1**	**1**	**494-516**	**R.SSSEKIHSNEISSDSIGVLGYQK.T**	
**227**	**1**	**1233.6000**	**-2.73**	**2**	**29.4**	**39.7**	**1**	**494-516**	**R.SSSEKIHSNEISSDSIGVLGYQK.T**	**Deamidated: 22**
**257**	**2**	**974.0000**	**5.61**	**2**	**31.2**	**79.2**	**0**	**499-516**	**K.IHSNEISSDSIGVLGYQK.T**	
**357**	**1**	**549.1000**	**85.04**	**4**	**36.9**	**96.6**	**3**	**517-535**	**K.TVDHTKVNSKLSLFFEIKS.-**	
**496**	**2**	**542.4000**	**169.88**	**2**	**44.8**	**47.6**	**1**	**527-535**	**K.LSLFFEIKS.-**	

Project Info
Name: CRM

Sample Info & Protocols
Name:CRMstd: GluC digest

Protein 1: toxin CRM197
Accession: gi|224021|prf||1007216A
Database: allCRM_Kolarich
Seq. Coverage [%]: 48.00 %
Modification(s): Carbamidomethyl, Oxidation, Deamidated

Score: 3240.78
MW [kDa]: 58.40
pI: 5.83
No. of Peptides: 60

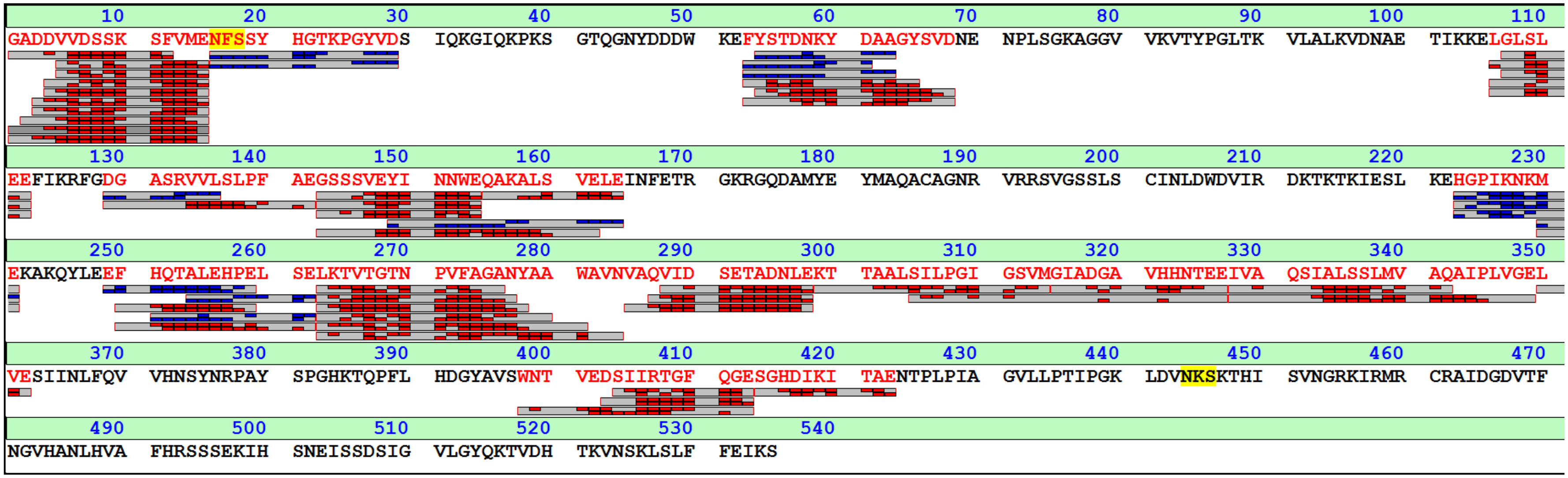

Cmpd.	No. of Cmpds.	*m/z* meas.	Δ *m/z* [ppm]	z	Rt [min]	Score	P	Range	Sequence	Modification
235	2	613.9070	206.25	2	40.9	38.5	0	1-12	-.GADDVVDSSKSF.V	
278	22	801.3920	47.91	2	48.1	78.3	0	1-15	-.GADDVVDSSKSFVME.N	Oxidation: 14
306	12	793.4030	59.05	2	53.7	96.9	0	1-15	-.GADDVVDSSKSFVME.N	
268	2	772.9480	136.02	2	46.3	70.9	0	2-15	G.ADDVVDSSKSFVME.N	Oxidation: 13

304	1	729.4290	140.04	2	53.3	36.7	0	3-15	A.DDVVDSSKSFVME.N	
272	2	737.4220	132.48	2	47.0	75.5	0	3-15	A.DDVVDSSKSFVME.N	Oxidation: 12
264	2	679.9080	142.91	2	45.7	74.1	0	4-15	D.DVVDSSKSFVME.N	Oxidation: 11
297	2	671.9280	170.59	2	52.1	23.7	0	4-15	D.DVVDSSKSFVME.N	
248	1	614.4190	193.84	2	42.7	35.5	0	5-15	D.VVDSSKSFVME.N	
211	3	622.4010	166.51	2	36.1	34.9	0	5-15	D.VVDSSKSFVME.N	Oxidation: 10
243	1	786.9100	73.84	2	42.2	28.9	0	16-29	E.NFSSYHGTKPGYVD.S	Deamidated: 1
207	3	786.4360	96.79	2	35.6	32.8	0	16-29	E.NFSSYHGTKPGYVD.S	
192	2	576.8600	197.95	2	33.3	30.5	0	53-61	E.FYSTDNKYD.A	
210	4	647.8930	169.89	2	35.8	33.4	0	53-63	E.FYSTDNKYDAA.G	
249	1	757.8990	97.19	2	42.8	39.8	0	53-65	E.FYSTDNKYDAAGY.S	
273	1	908.4090	21.97	2	47.0	22.8	0	53-68	E.FYSTDNKYDAAGYSVD.N	
157	2	574.3660	204.19	2	27.1	30.0	0	54-63	F.YSTDNKYDAA.G	
232	2	834.9200	78.06	2	40.5	73.0	0	54-68	F.YSTDNKYDAAGYSVD.N	
401	2	609.9620	236.22	2	66.7	61.9	0	106-116	E.LGLSLTEPLME.Q	Oxidation: 10
404	2	931.5360	83.09	2	67.1	96.3	0	106-122	E.LGLSLTEPLMEQVGTEE.F	Oxidation: 10
417	1	923.5320	76.72	2	69.0	74.8	0	106-122	E.LGLSLTEPLMEQVGTEE.F	
350	2	553.4160	253.19	2	60.0	32.8	0	107-116	L.GLSLTEPLME.Q	Oxidation: 9
373	2	874.9860	79.35	2	63.5	77.7	0	107-122	L.GLSLTEPLMEQVGTEE.F	Oxidation: 9
220	1	408.8580	307.45	2	37.3	28.8	0	129-136	G.DGASRVVL.S	
405	4	731.0030	153.48	2	67.5	40.2	0	129-142	G.DGASRVVLSLPFAE.G	
316	4	692.9020	140.86	2	55.6	54.0	0	143-154	E.GSSSVEYINNWE.Q	
333	3	693.4070	159.50	2	57.9	50.6	0	143-154	E.GSSSVEYINNWE.Q	Deamidated: 9
309	3	693.4160	172.48	2	54.7	44.9	0	143-154	E.GSSSVEYINNWE.Q	Deamidated: 10
361	2	1106.0740	38.11	2	61.7	79.1	0	143-162	E.GSSSVEYINNWEQAKALSVE.L	
379	2	954.0040	24.22	2	64.2	26.2	0	149-164	E.YINNWEQAKALSVELE.I	
250	4	544.4100	195.99	2	43.1	45.2	0	155-164	E.QAKALSVELE.I	
95	1	500.5600	113.31	4	19.4	77.2	0	223-240	E.HGPIKNKMSESPNKTVSE.E	Oxidation: 8
112	1	667.4220	137.92	3	21.8	22.9	0	223-240	E.HGPIKNKMSESPNKTVSE.E	Oxidation: 8; Deamidated: 13
102	2	532.8300	124.01	4	20.3	67.9	0	223-241	E.HGPIKNKMSESPNKTVSEE.K	Oxidation: 8
154	2	669.4370	207.52	2	26.7	31.7	0	230-241	K.MSESPNKTVSEE.K	
119	2	677.4070	164.53	2	22.8	41.1	0	230-241	K.MSESPNKTVSEE.K	Oxidation: 1
187	1	446.6360	210.05	3	32.5	52.3	0	249-259	E.EFHQTALEHPE.L	
168	9	604.8450	93.64	2	29.5	55.4	0	250-259	E.FHQTALEHPE.L	
234	6	769.4840	151.15	2	40.9	72.7	0	250-262	E.FHQTALEHPELSE.L	
195	2	695.9340	144.42	2	34.0	25.4	0	251-262	F.HQTALEHPELSE.L	
215	1	512.8340	162.03	2	36.6	24.9	0	254-262	T.ALEHPELSE.L	
280	2	688.4820	144.35	2	48.9	36.3	0	263-276	E.LKTVTGTNPVFAGA.N	
271	2	745.5020	131.34	2	47.0	65.2	0	263-277	E.LKTVTGTNPVFAGAN.Y	
305	1	827.0010	78.89	2	53.4	76.0	0	263-278	E.LKTVTGTNPVFAGANY.A	

**308**	**1**	**898.0060**	**36.89**	**2**	**54.3**	**59.9**	**0**	**263-280**	**E.LKTVTGTNPVFAGANYAA.W**	
**393**	**1**	**991.0450**	**32.76**	**2**	**65.4**	**73.2**	**0**	**263-281**	**E.LKTVTGTNPVFAGANYAAW.A**	
**398**	**1**	**1133.6380**	**486.49**	**2**	**66.2**	**89.2**	**0**	**263-284**	**E.LKTVTGTNPVFAGANYAAWAVN.V**	
**282**	**1**	**752.4520**	**119.23**	**2**	**49.5**	**103.3**	**0**	**285-298**	**N.VAQVIDSETADNLE.K**	
**259**	**1**	**667.4150**	**158.05**	**2**	**44.5**	**85.6**	**0**	**287-298**	**A.QVIDSETADNLE.K**	
**238**	**2**	**603.3830**	**170.34**	**2**	**41.4**	**52.0**	**0**	**288-298**	**Q.VIDSETADNLE.K**	
**394**	**1**	**788.0460**	**128.51**	**2**	**65.7**	**75.2**	**0**	**299-314**	**E.KTTAALSILPGIGSVM.G**	**Oxidation: 16**
**369**	**1**	**774.4770**	**556.67**	**3**	**62.8**	**24.9**	**0**	**305-327**	**L.SILPGIGSVMGIADGAVHHNTEE.I**	**Oxidation: 10**
**151**	**2**	**675.4300**	**181.17**	**2**	**26.5**	**44.6**	**0**	**315-327**	**M.GIADGAVHHNTEE.I**	
**399**	**1**	**774.0050**	**98.08**	**2**	**66.3**	**32.2**	**0**	**328-342**	**E.IVAQSIALSSLMVAQ.A**	**Oxidation: 12**
**441**	**1**	**1113.6920**	**60.77**	**2**	**71.3**	**77.2**	**0**	**328-349**	**E.IVAQSIALSSLMVAQAIPLVGE.L**	**Oxidation: 12**
**431**	**1**	**729.4720**	**140.95**	**2**	**70.2**	**62.8**	**0**	**350-362**	**E.LVDIGFAAYNFVE.S**	
**372**	**2**	**926.4970**	**53.73**	**2**	**63.1**	**48.6**	**0**	**398-413**	**S.WNTVEDSIIRTGFQGE.S**	
**274**	**2**	**611.9280**	**198.17**	**2**	**47.3**	**65.0**	**0**	**403-413**	**E.DSIIRTGFQGE.S**	
**245**	**1**	**554.4030**	**197.93**	**2**	**42.3**	**33.6**	**0**	**404-413**	**D.SIIRTGFQGE.S**	
**165**	**2**	**535.8690**	**170.83**	**2**	**28.5**	**41.2**	**0**	**414-423**	**E.SGHDIKITAE.N**	

32

Project Info

Name: CRM

Sample Info & Protocols

Name: CRMstd Glu-C/ Tryp digest

**Protein 1:**	toxin CRM197		
**Accession:**	gi|224021|prf||1007216A	**Score:**	7039.95
**Database:**	allCRM_Kolarich	**MW [kDa]:**	58.40
**Seq. Coverage [%]:**	84.70 %	**pI:**	5.83
		**No. of Peptides:**	99

Modification(s): Carbamidomethyl, Oxidation, Deamidated

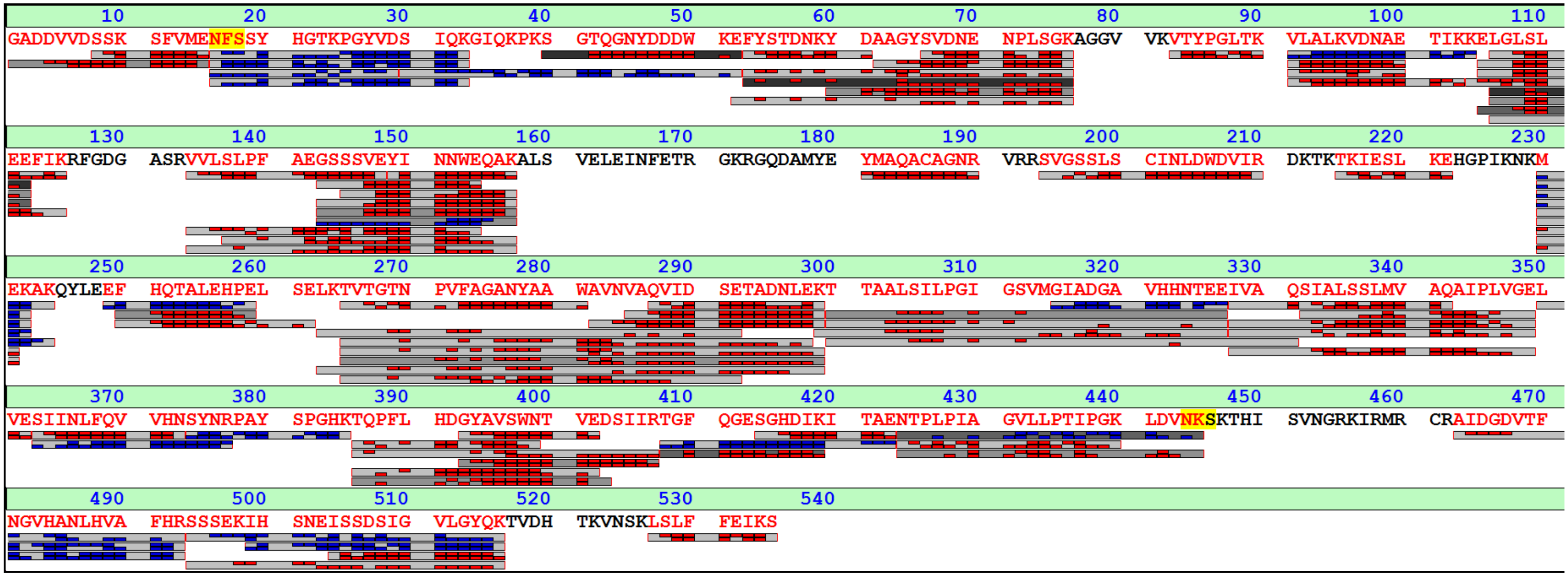

Cmpd.	No. of Cmpds.	*m/z* meas.	Δ *m/z* [ppm]	z	Rt [min]	Score	P	Range	Sequence	Modification
207	10	793.4140	72.91	2	44.8	93.1	0	1-15	-.GADDVVDSSKSFVME.N	
98	2	457.7210	6.48	2	28.3	51.3	0	8-15	D.SSKSFVME.N	
109	2	786.4200	76.44	2	29.7	34.2	0	16-29	E.NFSSYHGTKPGYVD.S	
137	13	676.7060	59.76	3	33.4	131.9	0	16-33	E.NFSSYHGTKPGYVDSIQK.G	
153	2	508.0390	82.69	4	35.1	73.7	0	16-33	E.NFSSYHGTKPGYVDSIQK.G	Deamidated: 1
141	1	508.0430	90.56	4	33.8	53.9	0	16-33	E.NFSSYHGTKPGYVDSIQK.G	Deamidated: 17
96	1	656.3640	58.94	4	28.2	69.4	0	30-52	D.SIQKGIQKPKSGTQGNYDDDWKE.F	
112	6	757.8180	16.97	2	30.0	69.7	0	40-52	K.SGTQGNYDDDWKE.F	
204	1	929.0840	362.61	3	44.1	81.3	0	52-76	K.EFYSTDNKYDAAGYSVDNENPLSGK.A	
97	2	576.7640	31.49	2	28.3	39.5	0	53-61	E.FYSTDNKYD.A	
208	13	885.7710	42.87	3	45.3	84.5	0	53-76	E.FYSTDNKYDAAGYSVDNENPLSGK.A	
201	1	886.0760	16.89	3	43.5	53.9	0	53-76	E.FYSTDNKYDAAGYSVDNENPLSGK.A	Deamidated: 6
185	2	900.4550	52.46	2	40.6	117.3	0	60-76	K.YDAAGYSVDNENPLSGK.A	
155	2	761.3820	25.45	2	36.0	76.9	0	62-76	D.AAGYSVDNENPLSGK.A	
103	1	580.8180	921.76	2	29.3	35.0	0	66-76	Y.SVDNENPLSGK.A	
146	12	439.7700	39.26	2	34.4	40.7	0	83-90	K.VTYPGLTK.V	
168	17	536.3450	72.97	2	38.5	82.3	0	91-100	K.VLALKVDNAE.T	
190	1	536.8220	44.95	2	41.2	26.2	0	91-100	K.VLALKVDNAE.T	Deamidated: 8
160	2	707.4590	56.24	2	36.6	88.6	0	91-103	K.VLALKVDNAETIK.K	
149	2	515.0190	85.64	3	34.8	52.6	0	91-104	K.VLALKVDNAETIKK.E	Deamidated: 8
264	1	730.4610	98.21	2	52.9	69.8	0	104-116	K.KELGLSLTEPLME.Q	
328	2	666.4220	120.38	2	63.3	65.7	0	105-116	K.ELGLSLTEPLME.Q	
303	1	674.4170	115.31	2	58.3	70.9	0	105-116	K.ELGLSLTEPLME.Q	Oxidation: 11
345	1	988.0670	85.58	2	65.4	116.6	0	105-122	K.ELGLSLTEPLMEQVGTEE.F	
293	2	609.9040	141.11	2	56.4	75.8	0	106-116	E.LGLSLTEPLME.Q	Oxidation: 10
305	2	931.5420	89.53	2	58.5	82.2	0	106-122	E.LGLSLTEPLMEQVGTEE.F	Oxidation: 10
336	2	923.5280	72.39	2	64.3	81.3	0	106-122	E.LGLSLTEPLMEQVGTEE.F	
314	2	1125.5940	10.37	2	59.8	64.7	0	106-125	E.LGLSLTEPLMEQVGTEEFIK.R	Oxidation: 10
140	2	525.8140	70.49	2	33.6	50.8	0	117-125	E.QVGTEEFIK.R	
321	1	760.9670	93.61	2	61.0	102.7	0	134-148	R.VVLSLPFAEGSSSVE.Y	
356	1	1170.5870	11.66	2	67.0	82.3	0	134-154	R.VVLSLPFAEGSSSVEYINNWE.Q	
340	2	1334.1980	21.98	2	64.7	110.2	0	134-157	R.VVLSLPFAEGSSSVEYINNWEQAK.A	
280	2	1178.5430	-12.92	2	54.9	107.0	0	137-157	L.SLPFAEGSSSVEYINNWEQAK.A	

212	2	692.8770	104.77	2	46.7	64.9	0	143-154	E.GSSSVEYINNWE.Q	
197	12	856.4400	47.01	2	42.7	94.7	0	143-157	E.GSSSVEYINNWEQAK.A	
173	2	856.9190	31.80	2	39.0	70.3	0	143-157	E.GSSSVEYINNWEQAK.A	Deamidated: 13
173	2	856.9190	31.80	2	39.0	88.0	0	143-157	E.GSSSVEYINNWEQAK.A	Deamidated: 9
181	1	784.3830	12.76	2	40.0	60.2	0	145-157	S.SSVEYINNWEQAK.A	
118	2	583.2810	-7.65	2	30.9	73.4	0	149-157	E.YINNWEQAK.A	
51	2	571.2430	-7.88	2	21.5	81.7	0	181-190	E.YMAQACAGNR.V	Carbamidomethyl: 6
312	2	961.0480	73.02	2	59.6	88.0	0	194-210	R.SVGSSLSCINLDWDVIR.D	Carbamidomethyl: 8
52	2	474.2720	-4.29	2	21.8	34.7	0	215-222	K.TKIESLKE.H	
58	2	604.7620	-24.49	2	22.5	66.4	0	230-240	K.MSESPNKTVSE.E	
48	1	613.2940	45.21	2	21.1	66.7	0	230-240	K.MSESPNKTVSE.E	Oxidation: 1; Deamidated: 6
45	3	677.3070	16.88	2	20.6	69.0	0	230-241	K.MSESPNKTVSEE.K	Oxidation: 1
68	4	669.3340	53.63	2	24.1	61.1	0	230-241	K.MSESPNKTVSEE.K	
38	1	494.5630	-2.94	3	19.6	92.3	0	230-242	K.MSESPNKTVSEEK.A	Oxidation: 1
46	5	489.2300	-5.76	3	20.8	79.2	0	230-242	K.MSESPNKTVSEEK.A	
53	2	489.5620	2.40	3	21.8	76.9	0	230-242	K.MSESPNKTVSEEK.A	Deamidated: 6
33	2	555.5980	-21.92	3	19.1	70.3	0	230-244	K.MSESPNKTVSEEKAK.Q	
47	1	417.1950	-25.06	4	20.9	48.1	0	230-244	K.MSESPNKTVSEEKAK.Q	Deamidated: 6
92	1	446.5280	-31.81	3	27.7	38.7	0	249-259	E.EFHQTALEHPE.L	
75	15	604.7740	-23.76	2	24.9	63.3	0	250-259	E.FHQTALEHPE.L	
138	2	769.4110	56.26	2	33.3	55.6	0	250-262	E.FHQTALEHPELSE.L	
360	1	1036.8920	351.51	3	67.7	34.6	0	263-292	E.LKTVTGTNPVFAGANYAAWAVNV AQVIDSE.T	Deamidated: 22
366	1	1293.6310	236.54	3	68.1	79.9	0	263-299	E.LKTVTGTNPVFAGANYAAWAVNV AQVIDSETADNLEK.T	
307	1	870.4820	67.76	2	58.8	91.7	0	265-281	K.TVTGTNPVFAGANYAAW.A	
370	2	1433.6900	337.48	2	68.9	91.8	0	265-292	K.TVTGTNPVFAGANYAAWAVNVAQ VIDSE.T	
377	2	1170.6250	334.34	3	69.9	112.2	0	265-298	K.TVTGTNPVFAGANYAAWAVNVAQ VIDSETADNLE.K	
395	5	1213.2940	27.96	3	73.0	50.8	0	265-299	K.TVTGTNPVFAGANYAAWAVNVAQ VIDSETADNLEK.T	Deamidated: 20
372	14	1213.3080	309.94	3	69.4	112.9	0	265-299	K.TVTGTNPVFAGANYAAWAVNVAQ VIDSETADNLEK.T	
235	2	958.5440	62.60	2	49.4	142.6	0	282-299	W.AVNVAQVIDSETADNLEK.T	
157	2	816.4200	12.53	2	36.4	120.9	0	285-299	N.VAQVIDSETADNLEK.T	
135	2	731.3780	28.70	2	33.0	77.6	0	287-299	A.QVIDSETADNLEK.T	
304	1	963.8950	66.74	3	58.2	49.9	0	299-327	E.KTTAALSILPGIGSVMGIADGAV HHNTEE.I	
351	1	921.1940	66.92	3	66.6	40.2	0	300-327	K.TTAALSILPGIGSVMGIADGAVH HNTEE.I	
300	5	926.5010	39.94	3	57.7	63.2	0	300-327	K.TTAALSILPGIGSVMGIADGAVH	Oxidation: 15

									HNTEE.I	
352	1	1058.2700	51.90	3	67.0	25.0	0	300-331	K.TTAALSILPGIGSVMGIADGAVH HNTEEIVAQ.S	
63	3	450.5200	-46.30	3	23.3	60.6	0	315-327	M.GIADGAVHHNTEE.I	
287	1	774.0190	116.17	2	55.8	51.6	0	328-342	E.IVAQSIALSSLMVAQ.A	Oxidation: 12
359	2	1113.6500	23.05	2	67.5	117.3	0	328-349	E.IVAQSIALSSLMVAQAIPLVGE. L	Oxidation: 12
371	10	1105.6940	60.71	2	69.2	90.8	0	328-349	E.IVAQSIALSSLMVAQAIPLVGE. L	
380	2	1106.1780	53.45	2	70.7	46.7	0	328-349	E.IVAQSIALSSLMVAQAIPLVGE. L	Deamidated: 4
363	1	900.0730	78.01	2	67.9	41.9	0	332-349	Q.SIALSSLMVAQAIPLVGE.L	
347	2	729.4430	101.19	2	65.9	97.2	0	350-362	E.LVDIGFAAYNFVE.S	
302	2	642.4270	105.92	2	58.1	72.1	0	363-373	E.SIINLFQVVHN.S	
266	2	602.0390	84.39	3	53.3	65.1	0	363-377	E.SIINLFQVVHNSYNR.P	
40	2	459.5570	-10.25	3	19.8	50.4	0	374-385	N.SYNRPAYSPGHK.T	
227	2	817.9540	86.16	2	48.5	39.3	0	386-399	K.TQPFLHDGYAVSWN.T	
268	14	982.5080	45.93	2	53.4	63.0	0	386-402	K.TQPFLHDGYAVSWNTVE.D	
257	7	1040.0050	27.55	2	51.7	52.7	0	386-403	K.TQPFLHDGYAVSWNTVED.S	
309	16	850.1520	76.37	3	59.1	83.7	0	386-407	K.TQPFLHDGYAVSWNTVEDSIIR. T	
214	2	563.3080	77.77	2	47.0	65.4	0	393-402	D.GYAVSWNTVE.D	
282	4	855.5220	109.53	2	55.2	112.9	0	393-407	D.GYAVSWNTVEDSIIR.T	
60	4	638.2940	-12.28	2	22.9	88.8	0	408-419	R.TGFQGESGHDIK.I	
144	2	563.9610	35.81	3	34.0	93.8	0	408-423	R.TGFQGESGHDIKITAE.N	
73	2	535.7930	28.99	2	24.5	67.9	0	414-423	E.SGHDIKITAE.N	
325	6	851.0610	49.24	2	62.5	77.2	0	424-440	E.NTPLPIAGVLLPTIPGK.L	
318	12	1135.7370	52.17	2	60.5	84.1	0	424-445	E.NTPLPIAGVLLPTIPGKLDVNK. S	
317	1	757.8160	44.52	3	60.2	33.0	0	424-445	E.NTPLPIAGVLLPTIPGKLDVNK. S	Deamidated: 21
124	2	460.2610	65.25	3	31.7	36.7	0	463-474	R.AIDGDVTFCRPK.S	Carbamidomethyl: 9
177	1	416.0230	21.59	5	39.6	44.1	0	475-493	K.SPVYVGNGVHANLHVAFHR.S	Deamidated: 7, 12
192	8	519.5670	91.05	4	41.5	91.5	0	475-493	K.SPVYVGNGVHANLHVAFHR.S	Deamidated: 7
158	6	519.3010	52.58	4	36.7	89.2	0	475-493	K.SPVYVGNGVHANLHVAFHR.S	
169	2	822.7740	43.75	3	38.5	73.1	0	494-516	R.SSSEKIHSNEISSDSIGVLGYQK .T	Deamidated: 9
170	2	617.3310	440.29	4	38.5	58.4	0	494-516	R.SSSEKIHSNEISSDSIGVLGYQK .T	
178	2	649.6870	33.17	3	39.8	137.7	0	499-516	K.IHSNEISSDSIGVLGYQK.T	
202	16	683.8990	50.78	2	43.8	133.8	0	504-516	E.ISSDSIGVLGYQK.T	
286	2	542.3730	120.09	2	55.7	46.8	0	527-535	K.LSLFFEIKS.-	

Project Info

Name: CRM

Sample Info & Protocols

Name: CRM_std_AspC_GluC

Protein 1: Diphtheria toxin OS=Corynephage beta PE=1 SV=2 no signal peptide

**Accession:**	sp|P00588|DTX_CORBE_NO_SIGNAL	**Score:**	5432.55
**Database:**	allCRM_Kolarich	**MW [kDa]:**	58.30
**Seq. Coverage [%]:**	71.20 %	**pI:**	5.91
		**No. of Peptides:**	98

Modification(s): Carbamidomethyl, Oxidation, Deamidated

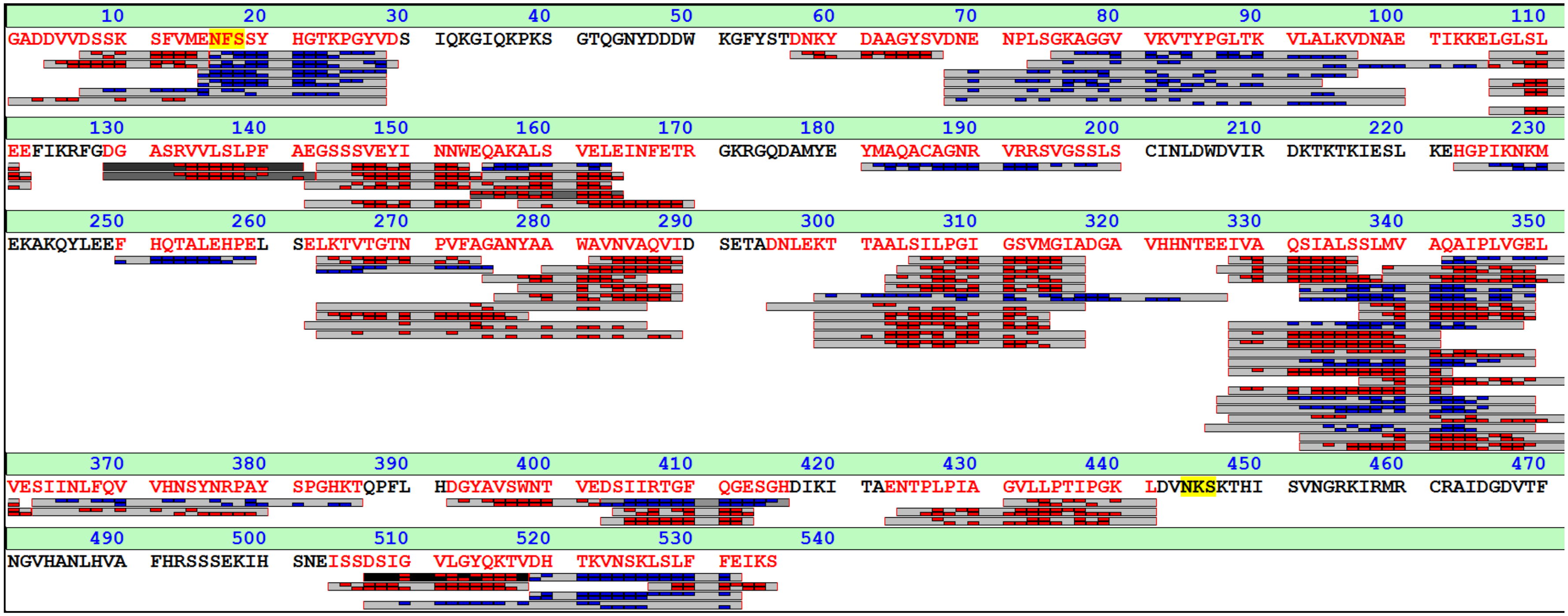

Cmpd.	No. of Cmpds.	*m/z* meas.	Δ *m/z* [ppm]	z	Rt [min]	Score	P	Range	Sequence	Modification
250	2	756.5570	-55.04	4	48.7	40.9	0	1-28	-.GADDVVDSSKSFVMENFSSYHGT KPGYV.D	
150	2	679.7580	-77.74	2	35.9	35.4	0	4-15	D.DVVDSSKSFVME.N	Oxidation: 11
74	1	523.7380	972.87	2	26.9	24.9	0	7-15	V.DSSKSFVME.N	Oxidation: 8
185	1	621.7710	379.63	4	39.9	31.2	0	7-28	V.DSSKSFVMENFSSYHGTKPGYV. D	Oxidation: 8
133	1	529.5710	-8.63	3	34.2	35.1	0	15-28	M.ENFSSYHGTKPGYV.D	Deamidated: 2
100	1	529.1990	-91.77	3	30.6	88.2	0	15-28	M.ENFSSYHGTKPGYV.D	
88	8	486.1920	-85.08	3	28.9	55.5	0	16-28	E.NFSSYHGTKPGYV.D	
87	1	524.5330	-81.37	3	28.7	41.4	0	16-29	E.NFSSYHGTKPGYVD.S	
121	1	601.7360	-56.24	2	32.8	50.1	0	57-67	T.DNKYDAAGYSV.D	
243	1	657.6040	-17.53	4	47.3	66.2	0	68-93	V.DNENPLSGKAGGVVKVTYPGLTK VLA.L	
471	2	594.3620	31.35	5	73.3	42.4	0	68-96	V.DNENPLSGKAGGVVKVTYPGLTK VLALKV.D	
455	3	680.3480	256.87	5	71.5	22.5	0	68-100	V.DNENPLSGKAGGVVKVTYPGLTK VLALKVDNAE.T	
274	1	680.3820	17.51	5	51.7	23.6	0	68-100	V.DNENPLSGKAGGVVKVTYPGLTK VLALKVDNAE.T	Deamidated: 2
244	1	572.1940	43.75	6	47.8	29.9	0	73-105	P.LSGKAGGVVKVTYPGLTKVLALK VDNAETIKKE.L	Deamidated: 26
217	1	440.4640	-32.96	5	44.5	51.7	0	75-96	S.GKAGGVVKVTYPGLTKVLALKV. D	
336	1	537.3080	16.39	2	58.6	36.0	0	106-115	E.LGLSLTEPLM.E	
306	4	545.2890	-14.03	2	55.7	62.2	0	106-115	E.LGLSLTEPLM.E	Oxidation: 10
325	2	601.8410	34.08	2	57.6	73.0	0	106-116	E.LGLSLTEPLME.Q	
296	6	609.8060	-19.59	2	54.0	57.5	0	106-116	E.LGLSLTEPLME.Q	Oxidation: 10
301	2	931.3740	-90.83	2	54.7	73.3	0	106-122	E.LGLSLTEPLMEQVGTEE.F	Oxidation: 10
112	2	574.7320	-49.81	2	32.0	47.0	0	112-121	T.EPLMEQVGTE.E	Oxidation: 4
117	3	639.2310	-79.66	2	32.6	61.2	0	112-122	T.EPLMEQVGTEE.F	Oxidation: 4
303	5	666.3360	-50.31	2	55.1	44.7	0	129-141	G.DGASRVVLSLPFA.E	
298	2	730.9490	79.60	2	54.3	47.0	0	129-142	G.DGASRVVLSLPFAE.G	
233	2	692.7960	-12.14	2	46.2	45.2	0	142-153	A.EGSSSVEYINNW.E	
226	2	757.2610	-85.45	2	45.3	52.1	0	142-154	A.EGSSSVEYINNWE.Q	
225	2	628.2510	-51.12	2	45.1	66.3	0	143-153	E.GSSSVEYINNW.E	
214	1	692.7700	-49.67	2	44.2	65.5	0	143-154	E.GSSSVEYINNWE.Q	
161	2	544.2830	-37.33	2	37.3	62.9	0	154-163	W.EQAKALSVEL.E	
147	2	608.7860	-63.43	2	35.7	61.4	0	154-164	W.EQAKALSVELE.I	
146	2	479.7600	-45.91	2	35.4	31.6	0	155-163	E.QAKALSVEL.E	
129	2	544.2710	-59.38	2	33.9	52.7	0	155-164	E.QAKALSVELE.I	

269	2	760.8530	-63.59	2	50.6	75.5	0	158-170	K.ALSVELEINFETR.G	
73	2	547.2370	-59.00	4	27.0	78.7	0	181-200	E.YMAQACAGNRVRRSVGSSLS.C	Carbamidomethyl: 6; Oxidation: 2
23	1	400.5910	-32.66	5	21.9	30.6	0	223-240	E.HGPIKNKMSESPNKTVSE.E	Oxidation: 8
58	2	403.4780	-123.92	3	25.2	38.7	0	250-259	E.FHQTALEHPE.L	
335	1	855.3950	-56.08	3	58.7	56.2	0	262-286	S.ELKTVTGTNPVFAGANYAAWAVNVA.Q	
155	2	624.3100	-69.42	2	36.7	32.3	0	263-274	E.LKTVTGTNPVFA.G	
153	2	652.8080	-85.89	2	36.5	53.6	0	263-275	E.LKTVTGTNPVFAG.A	
192	1	826.8580	-94.04	2	41.0	72.2	0	263-278	E.LKTVTGTNPVFAGANY.A	
324	1	812.3710	-71.11	3	57.3	26.1	0	263-286	E.LKTVTGTNPVFAGANYAAWAVNVA.Q	
378	1	926.1330	324.62	3	63.9	53.5	0	263-289	E.LKTVTGTNPVFAGANYAAWAVNVAQVI.D	
253	2	603.7750	-39.31	2	49.0	31.7	0	275-286	A.GANYAAWAVNVA.Q	
366	1	745.3500	-58.40	2	62.3	46.7	0	276-289	G.ANYAAWAVNVAQVI.D	
345	1	652.8350	-28.36	2	59.8	28.6	0	278-289	N.YAAWAVNVAQVI.D	
308	2	535.8020	-2.41	2	55.8	57.2	0	280-289	A.AWAVNVAQVI.D	
202	2	407.2090	-88.59	2	42.7	55.6	0	282-289	W.AVNVAQVI.D	
362	1	763.0420	-48.02	3	62.0	53.1	0	295-317	A.DNLEKTTAALSILPGIGSVMGIA.D	Oxidation: 20
305	2	779.9120	-45.24	2	55.6	57.0	0	299-314	E.KTTAALSILPGIGSVM.G	
267	2	787.8760	-87.24	2	50.4	66.5	0	299-314	E.KTTAALSILPGIGSVM.G	Oxidation: 16
359	2	900.4430	-83.96	2	61.6	53.3	0	299-317	E.KTTAALSILPGIGSVMGIA.D	
318	7	908.4540	-68.31	2	57.0	80.2	0	299-317	E.KTTAALSILPGIGSVMGIA.D	Oxidation: 16
232	4	727.3390	296.30	4	46.2	75.9	0	299-327	E.KTTAALSILPGIGSVMGIADGAVHHNTEE.I	Oxidation: 16
387	1	699.8820	-32.70	2	65.0	45.5	0	303-317	A.ALSILPGIGSVMGIA.D	
355	1	707.8650	-52.75	2	61.1	63.8	0	303-317	A.ALSILPGIGSVMGIA.D	Oxidation: 12
353	1	672.3740	-14.56	2	60.7	52.0	0	304-317	A.LSILPGIGSVMGIA.D	Oxidation: 11
304	10	615.8150	-43.45	2	55.3	65.7	0	305-317	L.SILPGIGSVMGIA.D	Oxidation: 10
398	2	828.7240	-68.02	3	65.9	23.3	0	326-349	T.EEIVAQSIALSSLMVAQAIPLVGE.L	Oxidation: 14
211	2	515.7800	-24.41	2	43.8	20.9	0	327-336	E.EIVAQSIALS.S	
416	2	780.3870	-60.91	3	67.5	59.5	0	327-349	E.EIVAQSIALSSLMVAQAIPLVGE.L	
394	2	785.7310	-44.77	3	65.6	34.4	0	327-349	E.EIVAQSIALSSLMVAQAIPLVGE.L	Oxidation: 13
182	2	451.2480	-51.62	2	39.5	47.1	0	328-336	E.IVAQSIALS.S	
266	2	551.2880	-74.98	2	50.4	69.8	0	328-338	E.IVAQSIALSSL.M	
307	5	709.8770	-32.12	2	55.8	104.5	0	328-341	E.IVAQSIALSSLMVA.Q	Oxidation: 12
342	2	701.8530	-70.30	2	59.6	87.6	0	328-341	E.IVAQSIALSSLMVA.Q	

279	1	773.9040	-32.42	2	52.0	107.8	0	328-342	E.IVAQSIALSSLMVAQ.A	Oxidation: 12
327	1	765.8940	-49.13	2	57.7	94.9	0	328-342	E.IVAQSIALSSLMVAQ.A	
405	2	694.3520	-77.97	3	66.4	28.3	0	328-348	E.IVAQSIALSSLMVAQAIPLVG.E	
391	11	742.7220	-40.36	3	65.4	72.1	0	328-349	E.IVAQSIALSSLMVAQAIPLVGE.L	Oxidation: 12
411	4	737.3890	-42.50	3	67.1	61.6	0	328-349	E.IVAQSIALSSLMVAQAIPLVGE.L	
420	2	813.4940	30.15	3	67.7	42.0	0	328-351	E.IVAQSIALSSLMVAQAIPLVGELV.D	Oxidation: 12
389	2	600.3470	15.63	3	65.1	60.0	0	332-349	Q.SIALSSLMVAQAIPLVGE.L	
381	2	605.6640	-8.68	3	64.4	47.2	0	332-349	Q.SIALSSLMVAQAIPLVGE.L	Oxidation: 8
425	1	1006.0260	-52.71	2	68.1	28.3	0	332-351	Q.SIALSSLMVAQAIPLVGELV.D	
409	1	1014.0270	-48.80	2	66.7	101.5	0	332-351	Q.SIALSSLMVAQAIPLVGELV.D	Oxidation: 8
331	1	664.3820	20.86	2	58.1	70.3	0	337-349	S.SLMVAQAIPLVGE.L	
294	2	672.3510	-21.71	2	53.7	86.5	0	337-349	S.SLMVAQAIPLVGE.L	Oxidation: 3
399	1	770.4180	-34.24	2	65.9	70.6	0	337-351	S.SLMVAQAIPLVGELV.D	
235	5	572.2690	-67.36	2	46.7	42.5	0	339-349	L.MVAQAIPLVGE.L	Oxidation: 1
361	2	678.3860	3.26	2	61.8	58.3	0	339-351	L.MVAQAIPLVGELV.D	Oxidation: 1
337	2	519.8090	-8.32	2	59.0	59.0	0	342-351	A.QAIPLVGELV.D	
356	2	558.7690	-4.75	2	61.4	52.9	0	352-361	V.DIGFAAYNFV.E	
349	3	623.2950	3.28	2	60.1	73.1	0	352-362	V.DIGFAAYNFVE.S	
277	2	712.3680	-6.46	3	51.9	44.3	0	363-380	E.SIINLFQVVHNSYNRPAY.S	
210	1	549.2990	19.97	5	43.7	28.3	0	363-386	E.SIINLFQVVHNSYNRPAYSPGHKT.Q	
271	4	620.7400	-60.68	2	51.3	66.2	0	392-402	H.DGYAVSWNTVE.D	
186	15	611.7770	-48.64	2	40.1	78.2	0	403-413	E.DSIIRTGFQGE.S	
120	3	501.8550	-111.71	3	32.9	89.2	0	403-416	E.DSIIRTGFQGESGH.D	
125	1	554.2680	-45.62	2	33.5	34.0	0	404-413	D.SIIRTGFQGE.S	
372	2	972.0280	-55.99	2	63.3	50.2	0	423-441	A.ENTPLPIAGVLLPTIPGKL.D	
369	3	907.5680	7.57	2	62.8	96.8	0	424-441	E.NTPLPIAGVLLPTIPGKL.D	
259	2	554.3430	-31.46	2	49.5	41.9	0	431-441	A.GVLLPTIPGKL.D	
201	2	783.8970	-32.30	2	42.5	77.6	0	504-518	E.ISSDSIGVLGYQKTV.D	
193	8	640.3950	72.99	2	41.3	50.1	0	507-518	S.DSIGVLGYQKTV.D	
260	1	585.8900	-39.17	5	49.4	24.1	0	507-532	S.DSIGVLGYQKTVDHTKVNSKLSLFFE.I	
191	1	555.5610	-117.47	3	40.8	77.5	0	519-532	V.DHTKVNSKLSLFFE.I	
213	1	417.1790	-92.33	4	44.1	99.4	0	519-532	V.DHTKVNSKLSLFFE.I	Deamidated: 6
282	2	542.3340	48.17	2	52.3	23.0	0	527-535	K.LSLFFEIKS.-	

Supplementary Figure S2: Sequence coverage calculated by ProteinScape based on the analysis of the proteolytic peptides obtained from unconjugated standard CRM_{197}. Examples of a Trypsin digest, Glu-C digest, Trypsin and Glu-C digest as well as Glu-C and Asp-N digest are given. The data was analysed by Mascot Server 2.3 using a custom data base containing all available diphtheria toxin and mutant sequences.

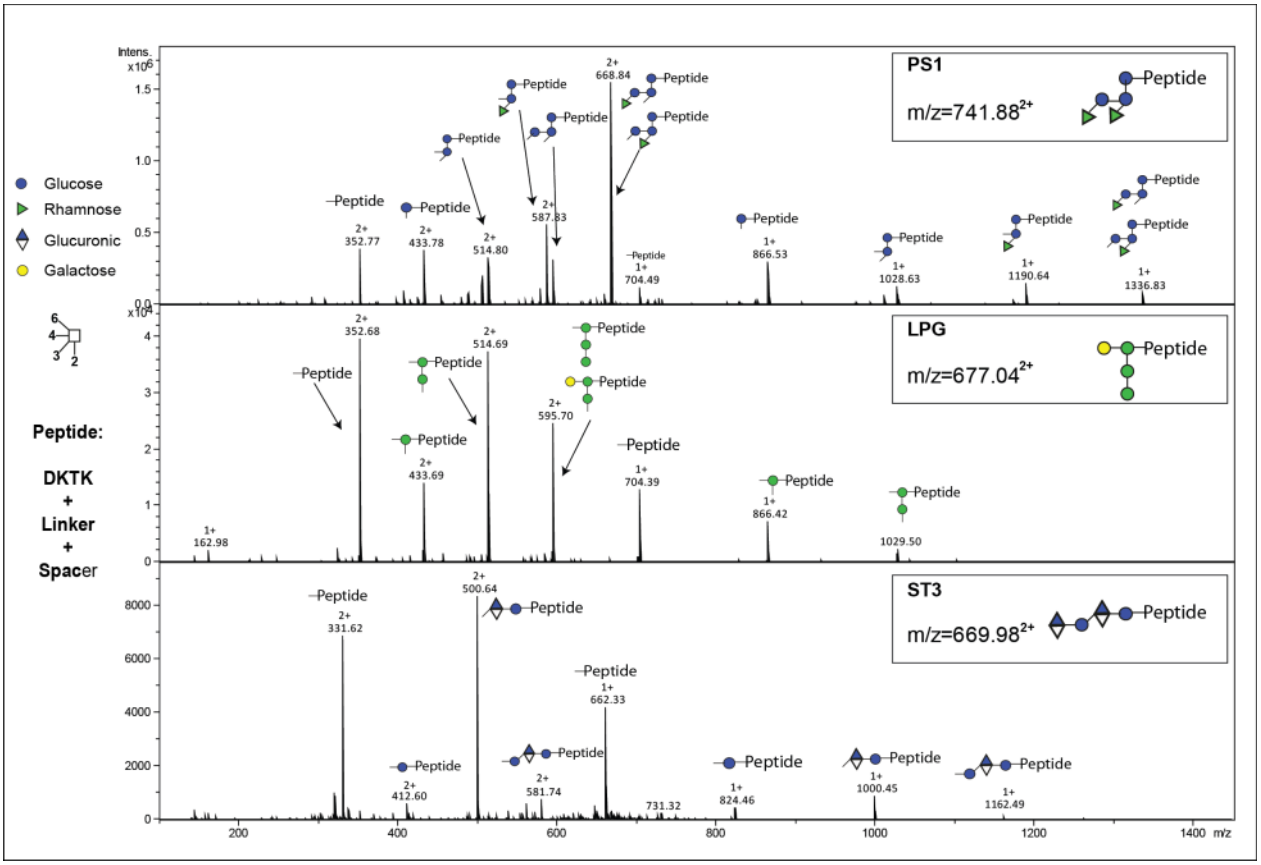

Supplementary Figure S3: CID fragment spectra of PS1, LPG and ST3 conjugated to K212 of the tryptic peptide ^{211}DKTK^{214} with K212 being conjugated. The doubly charged signal of each parent ion was selected for fragmentation. Characteristic fragment spectra were obtained with strong Y-ion abundance. LPG and PS 1 contained a C5 spacer whereas ST3 carried a C2 spacer, which accounts for the 42 m/z difference of the Y^0-ion containing only peptide, spacer and linker.

Supplementary Table S4: Peptides resulting from CNBr cleavage of CRM_{197} (NCBI Access No.: 1007216A). Lysine residues are indicated in bold. Grey labelled peptides do not carry any lysine residues or primary amines within the native folded protein and are therefore no substrates for conjugation. Due to its small mass peptide 197-182 was not detected by MALDI-TOF-MS.

Average $[M+H]^+$	Position	Artif. modification	Peptide sequence
1409.49	1-14	HSL: 14	**G**ADDVVDSS**K**SFVM
10976.15	15-115	HSL: 115	ENFSSYHGT**K**PGYVDSIQ**K**GIQ**K**P**K**SGTQGNYDDDW**K**EFYSTDN**K**YDAAGYSVDNENPLSG**K**AGGVV**K**VTYPGLT**K**VLAL**K**VDNAETI**KK**ELGLSLTEPLM
6975.68	116-178	HSL: 178	EQVGTEEFI**K**RFGDGASRVVLSLPFAEGSSSVEYINNWEQA**K**ALSVELEINFETRG**K**RGQDAM
557.58	179-182	HSL: 182	YEYM
5308.10	183-230	HSL: 230	AQACAGNRVRRSVGSSLSCINLDWDVIRD**K**T**K**T**K**IESL**K**EHGPI**K**N**K**M
8942.88	231-314	HSL: 314	SESPN**K**TVSEE**K**A**K**QYLEEFHQTALEHPELSEL**K**TVTGTNPVFAGANYAAWAVNVAQVIDSETADNLE**K**TTAALSILPGIGSVM
2516.77	315-339	HSL: 339	GIADGAVHHNTEEIVAQSIALSSLM
13076.88	340-459	HSL: 459	VAQAIPLVGELVDIGFAAYNFVESIINLFQVVHNSYNRPAYSPGH**K**TQPFLHDGYAVSWNTVEDSIIRTGFQGESGHDI**K**ITAENTPLPIAGVLLPTIPG**K**LDVN**K**S**K**THISVNGR**K**IRM
8417.51	460-535	-	RCRAIDGDVTFCRP**K**SPVYVGNGVHANLHVAFHRSSSE**K**IHSNEISSDSIGVLGYQ**K**TVDHT**K**VNS**K**LSLFFEI**K**S

Legend: HSL= homo serine lactone

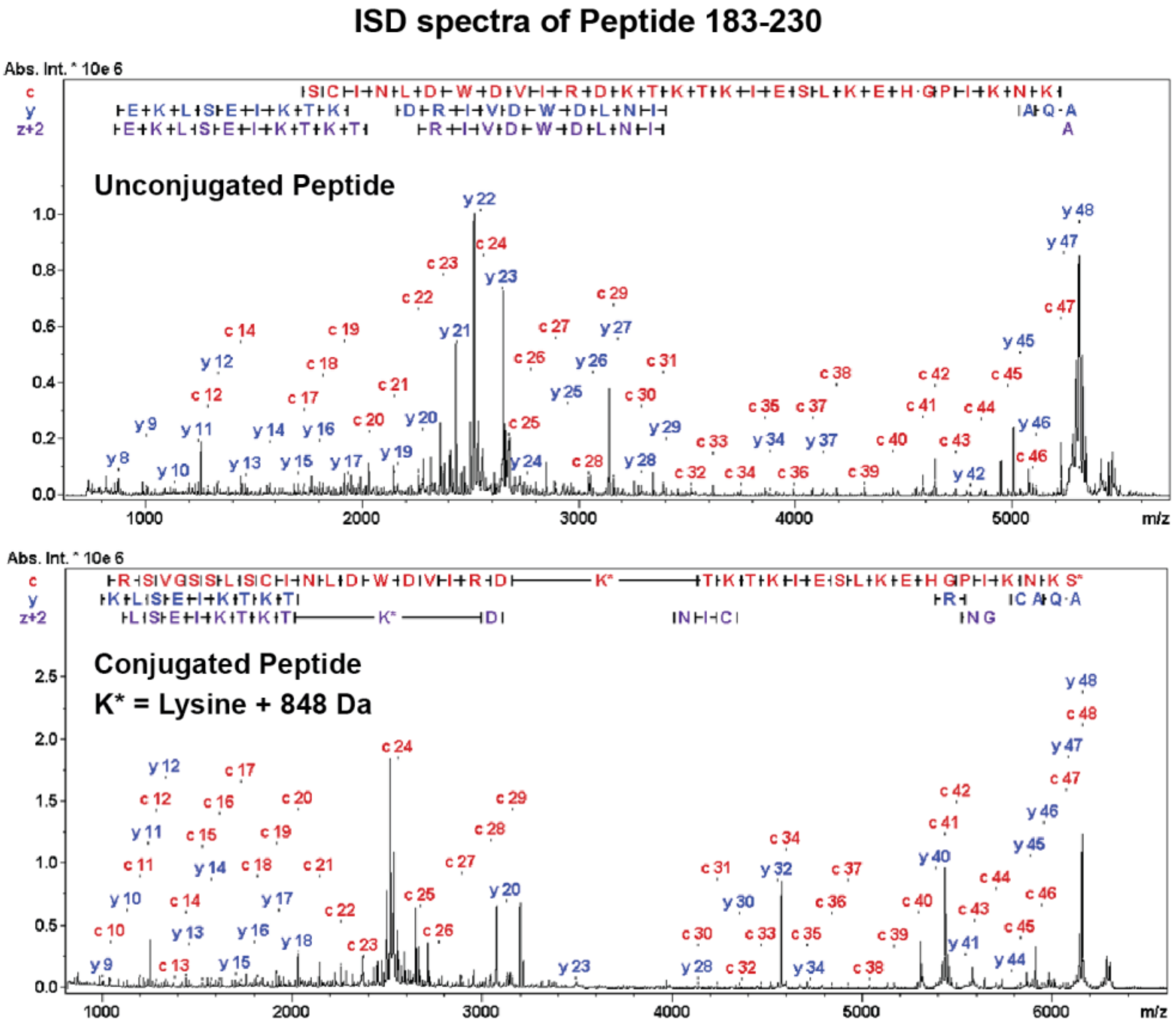

Supplementary Figure S5: ISD spectra of peptide 183-230 separated by C8. The c series in ST3 conjugated peptide (bottom) exhibited a shift of +848 Da from the c29- ion onwards compared to the unconjugated peptide (top), indicating conjugation to K212.

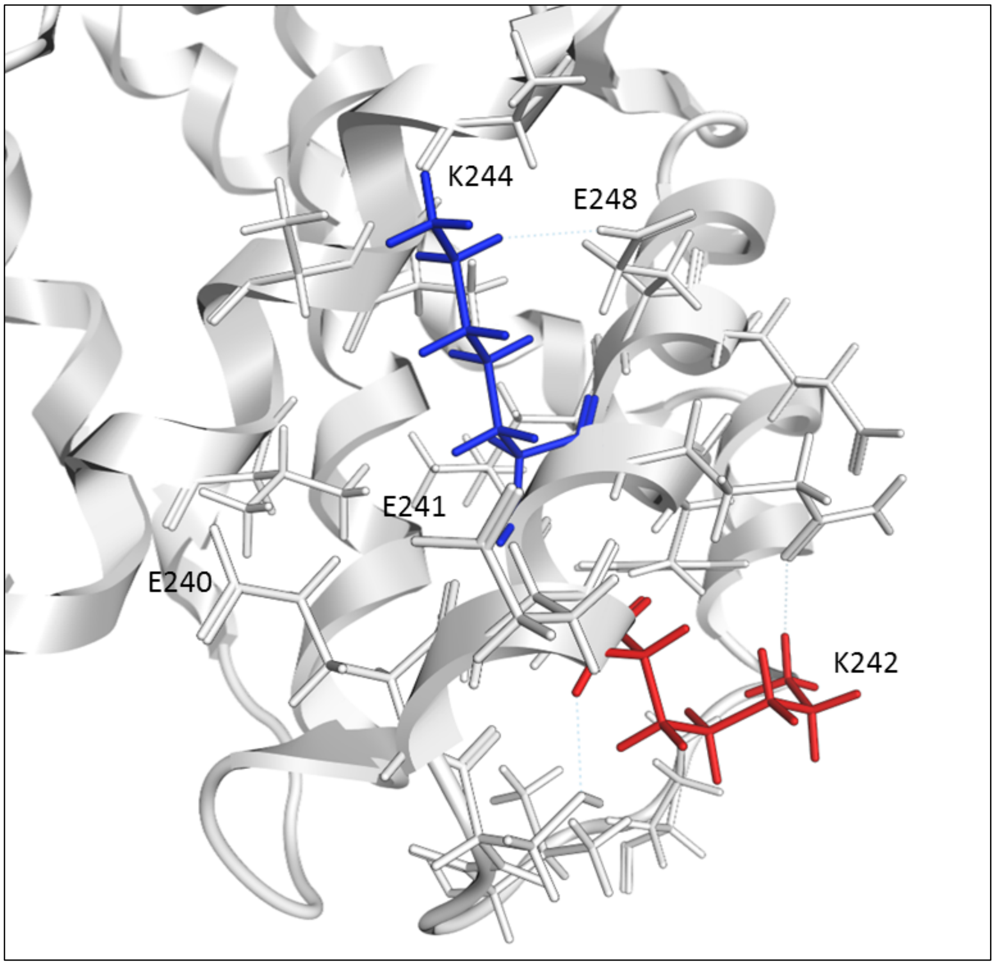

Supplementary Figure S6: Overview of glutamic acid residues that are located in close proximity to and K244 in the3D crystal structure of the CRM_{197} dimer (PDB entry: 4AE0). The formation of a salt bridge between E240, E241 or E248 and K244 may stabilize the positive charge on the lysine residue resulting in a reduced reactivity for glycan conjugation. In contrast, these glutamic acid residues are not close enough to K242 for a salt bridge formation furthermore no additional acidic residues are found in close enough proximity.

Supplementary material S7: Synthesis of GLC-PNP

General information:

Commercial grade solvents were used unless stated otherwise. Dry solvents were obtained from a Waters Dry Solvent System. Solvents for chromatography were distilled prior to use. Sensitive reactions were carried out in heat-dried glassware and under an argon atmosphere. Analytical thin layer chromatography (TLC) was performed on Kieselgel 60 F254 glass plates precoated with a 0.25 mm thickness of silica gel. Spots were visualized by staining with vanillin solution (6% (w/v) vanillin and 10% (v/v) sulfuric acid in 95% ethanol) or Hanessian's stain (5% (w/v) ammonium molybdate, 1% (w/v) cerium(II) sulfate and 10% (v/v) sulfuric acid in water). Silica column chromatography was performed on Fluka Kieselgel 60 (230-400 mesh).

^{1}H, ^{13}C and two-dimensional NMR spectra were measured with a Varian 400-MR, at 296 K. Chemical shifts (δ) are reported in parts per million (ppm) relative to the respective residual solvent peaks (cdcl$_3$: δ 7.27 in ^{1}H and 77.23 in ^{13}C NMR; CD_3OD: δ 3.31 in ^{1}H and 49.15 in ^{13}C NMR; D_2O: δ 4.80 in ^{1}H NMR). The following abbreviations are used to indicate peak multiplicities: *s* singlet; *d* doublet; *dd* doublet of doublets; *t* triplet; *dt* doublet of triplets; *q* quartet; *m* multiplet. Coupling constants (*J*) are reported in Hertz (Hz). Optical rotation (OR) measurements were carried out with a Schmidt & Haensch UniPol L1000 polarimeter at λ = 589 nm and a concentration (c) expressed in g/100 mL in the solvent noted in parentheses. High resolution mass spectrometry (HRMS) was performed at the Free University Berlin, Mass Spectrometry Core Facility, with an Agilent 6210 ESI-TOF mass spectrometer.

2,3-*O*-dibenzoyl-4,6-benzylidene-β-D-glucopyranosyl-(1→1)-(2-*N*-benzyl-*N*-benzyloxycarbonylamino) ethanol (X):

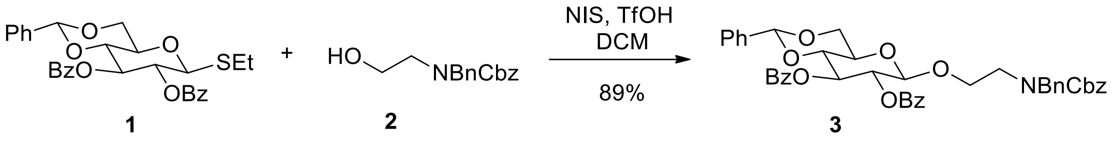

Thioglycoside donor **1** (6.0 g, 11.53 mmol) and C-2 linker **2** (dried azeotropically using toluene in rotary evaporator, 3.95 g, 13.83 mmol) were taken in dry DCM (100 mL). Added 5 g of MW dried 4 Å Molecular Sieve to it and stirred at room temperature for 15 min and then cooled to -10˚C. NIS (3.89 g, 17.29 mmol) and TfOH (0.15 mL, 1.73 mmol) were then added and the reaction mixture was stirred between -10 ˚C to -5 ˚C for 1 hr. Reaction mixture was then quenched with 10% aq. $Na_2S_2O_3$ solution (50 mL) and then extracted with EtOAc (25 mL x 3). The combined organic layer was washed with brine (10 mL), dried over anhyd. Na_2SO_4, filtered and concentrated. The crude product was purified on silica gel column chromatography using 20-30% EtOAc in hexanes yielding the desired product **3** as pale yellow gummy liquid which on high vacuum became a fluffy foam (7.60 g, 89%). $[\alpha]_D^{20}$ = -0.59° (c = 1.0, CH_2Cl_2) IR (thin film, cm^{-1}): v_{max} : 1728, 1699. ^{1}H NMR (400 MHz, $CDCl_3$) δ 7.95 (d, *J* = 8.1 Hz, 4H), 7.61 – 6.79 (m, 21H), 5.90 – 5.63 (m, 1H), 5.59 – 5.35 (m, 2H), 5.14 (s, 1H), 5.03 (dd, *J* = 33.7, 12.6 Hz, 1H), 4.78 (d, *J* = 7.7 Hz, 0.5H), 4.65 (d, *J* = 7.6 Hz, 0.5H), 4.48 – 4.23 (m, 3H), 4.13 – 3.48 (m, 5H), 3.47 – 3.23 (m, 2H). ^{13}C NMR (101 MHz, $cdcl_3$) δ 165.7, 165.4, 156.3, 156.2, 137.9, 136.9, 133.4, 133.2, 129.9, 129.5, 129.3, 129.1, 128.7, 128.5, 128.4, 128.3, 128.1, 127.8, 127.4, 127.2, 126.2, 101.9, 101.6, 78.9, 72.6, 72.1, 69.1, 68.7, 67.4, 67.2, 66.7,

51.7, 46.9, 45.8. HRMS (ESI): calculated for $C_{44}H_{41}NO_{10}$ $[M + Na]^+$, 766.2628, found 766.2657.

β-D-glucopyranosyl-(1→1)-2- aminoethanol (4):

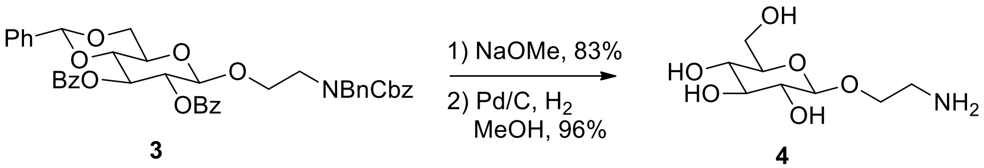

3 **4**

Compound **3** (0.1 g, 0,134 mmol) was taken in a mixture of MeOH and THF (5 mL/3 mL) and NaOMe in methanol (0.5 M, 1.613 mL, 0.807 mmol) was added and the reaction mixture stirred at room temperature for 20 h. Neutralized the reaction with Amberlite® 120 H^+ resin and stirred for 20 min. The solution was then filtered through a cotton plug and washed thoroughly with methanol and evaporated in vacuum. Ether (3 mL x 3) was then added and triturated to obtain the diol as an off-white solid after decantation of ether (0.060g, 83%). The diol (0.05 g, 0.093 mmol) was then taken in MeOH (3 mL) and Pd/C (~50 mg) was added and subjected to hydrogenolysis under atmospheric pressure of hydrogen at room temperature for 24 h. Filtered the reaction through PTFE hydrophobic filter and washed thoroughly with methanol (2 mL x 3) and water (2 mL x 2). Combined solvents were evaporated and the crude product was washed with ether (2 mL x 3) and DCM (2 mL x 3) and decanted. The residue was then dried under vacuum to obtain the compound **4** as a colorless fluffy solid (20 mg, 96%). ^1H NMR (400 MHz, d_2o) δ 4.59 (d, *J* = 7.9 Hz, 1H), 4.20 (dt, *J* = 11.7, 5.0 Hz, 1H), 4.07 – 3.94 (m, 2H), 3.80 (dd, *J* = 12.3, 5.8 Hz, 1H), 3.62 – 3.52 (m, 2H), 3.51 – 3.37 (m, 2H), 3.35 (t, *J* = 5.0 Hz, 2H). ^{13}C NMR (101 MHz, d_2o) δ 102.1, 75.9, 75.6, 73.0, 69.5, 65.8, 60.6, 39.4. HRMS (ESI): calculated for $C_8H_{17}NO_6$ $[M + H]^+$, 224.1134, found 224.1139.

Synthesis of *p*-nitrophenol activated ester 5:

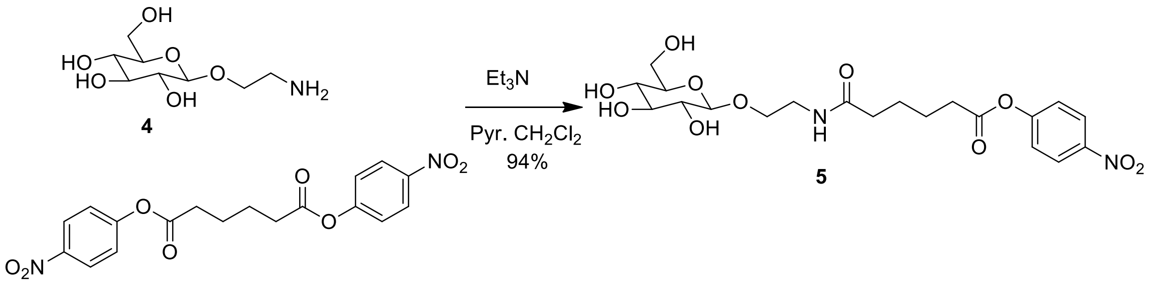

To a solution of compound **4** (0.002 g, 8.96 µmol) and bis(4-nitrophenyl)adipate (0.017 g, 0.045 mmol) in pyridine (0.4 mL) and DCM (0.2 mL) was added Et_3N (0.012 mL, 0.090 mmol) and the reaction mixture stirred at room temperature for 5 h. Diluted the reaction with toluene and removed the solvent under vacuum (3x). Purification by flash column chromatography using dichloromethane and methanol as eluent (5% to 20%) afforded the activated ester **5** as yellow oil (0.004 g, 94%). ^{1}H NMR (400 MHz, cd_3od) δ 8.36 – 8.21 (m, 2H), 7.46 – 7.25 (m, 2H), 4.28 (d, *J* = 7.8 Hz, 1H), 3.92 (ddd, *J* = 10.5, 6.3, 4.1 Hz, 1H), 3.87 (dd, *J* = 11.8, 1.7 Hz, 1H), 3.71 – 3.60 (m, 2H), 3.53 – 3.44 (m, 1H), 3.41 – 3.32 (m, 2H), 3.29 – 3.25 (m, 2H), 3.20 (dd, *J* = 9.1, 7.8 Hz, 1H), 2.68 (t, *J* = 7.0 Hz, 2H), 2.29 (t, *J* = 6.9 Hz, 2H), 1.90 – 1.64 (m, 4H). ^{13}C NMR (101 MHz, cd_3od) δ 172.7, 157.2, 126.2, 124.1, 104.6, 78.1, 78.0, 75.2, 71.7, 69.8, 62.8, 40.7, 37.1, 36.7, 34.7, 26.3, 25.3. HRMS (ESI): Calcd for $C_{20}H_{28}N_2O_{11}$ $[M+Na]^+$ 495.1591, found: 495.1609.

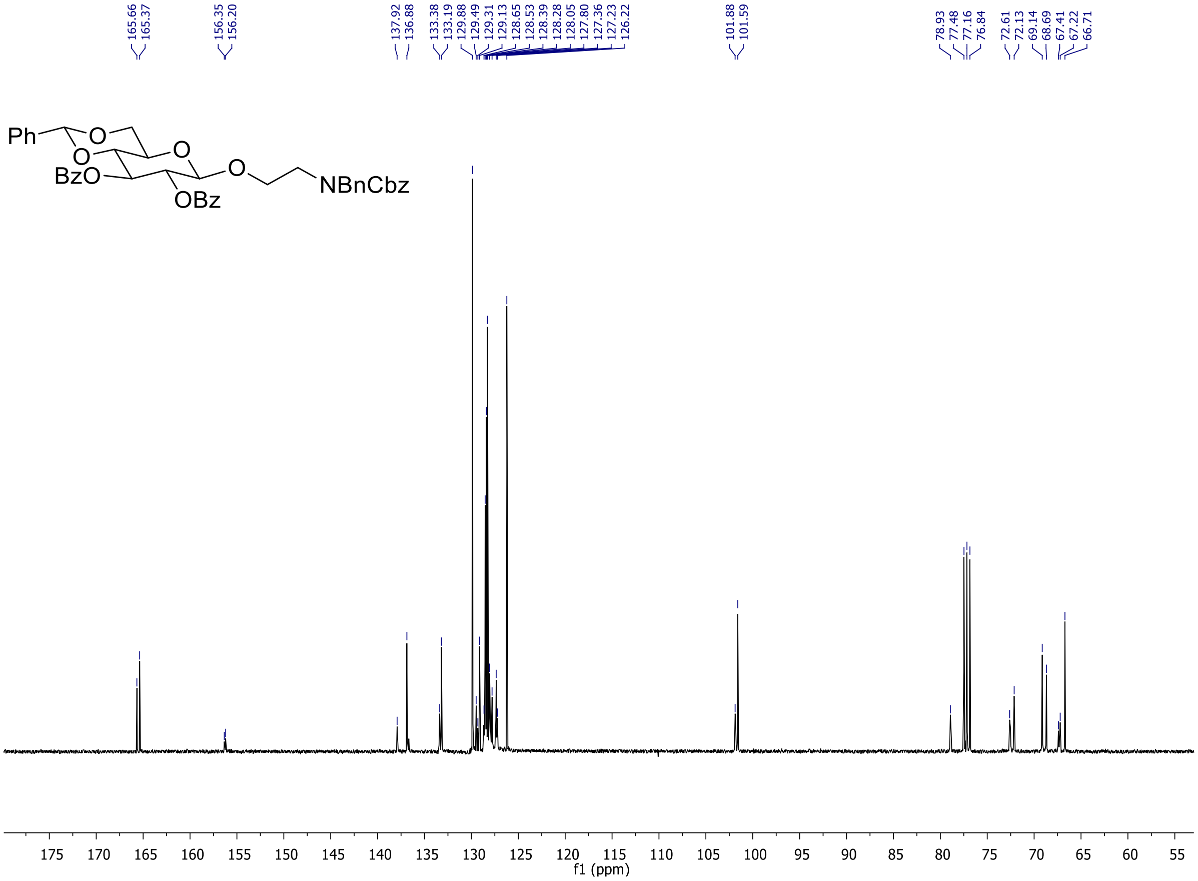
165.66
165.37
156.35
156.20
137.92
136.88
133.38
133.19
129.88
129.49
129.31
129.13
128.65
128.53
128.39
128.28
128.05
127.80
127.36
127.23
126.22
101.88
101.59
78.93
77.48
77.16
76.84
72.61
72.13
69.14
68.69
67.41
67.22
66.71
Ph
O
O
BzO
O
O
OBz
NBnCbz
175 170 165 160 155 150 145 140 135 130 125 120 115 110 105 100 95 90 85 80 75 70 65 60 55
f1 (ppm)

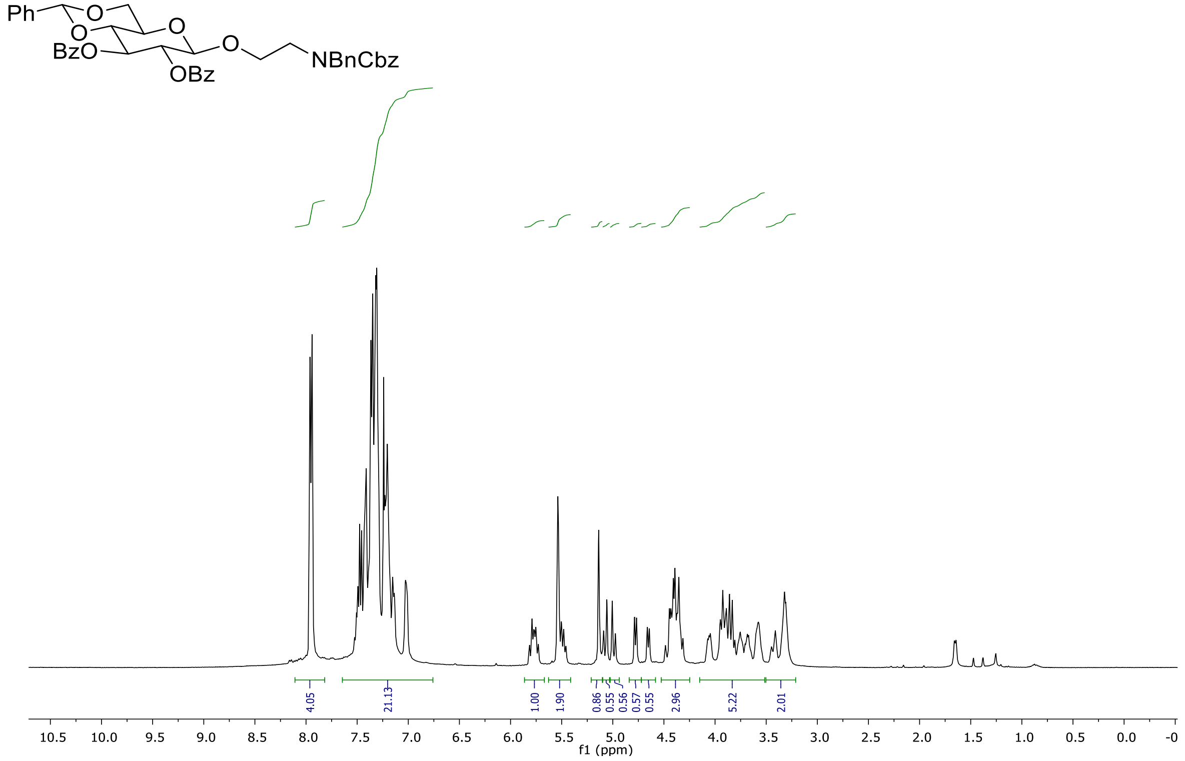
Ph
O
O
BzO
O
O
OBz
NBnCbz
4.05
21.13
1.00
1.90
0.86
0.55
0.56
0.57
0.55
2.96
5.22
2.01
10.5 10.0 9.5 9.0 8.5 8.0 7.5 7.0 6.5 6.0 5.5 5.0 4.5 4.0 3.5 3.0 2.5 2.0 1.5 1.0 0.5 0.0 -0
f1 (ppm)

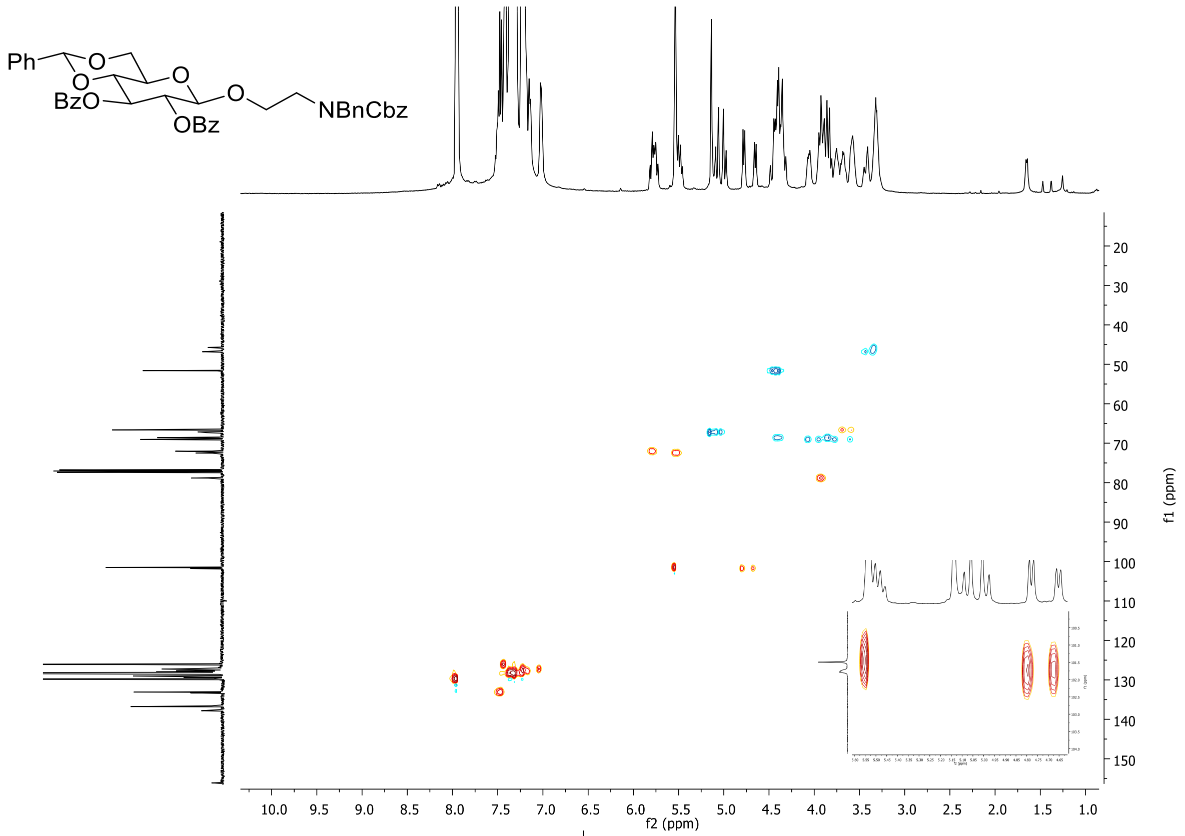

149

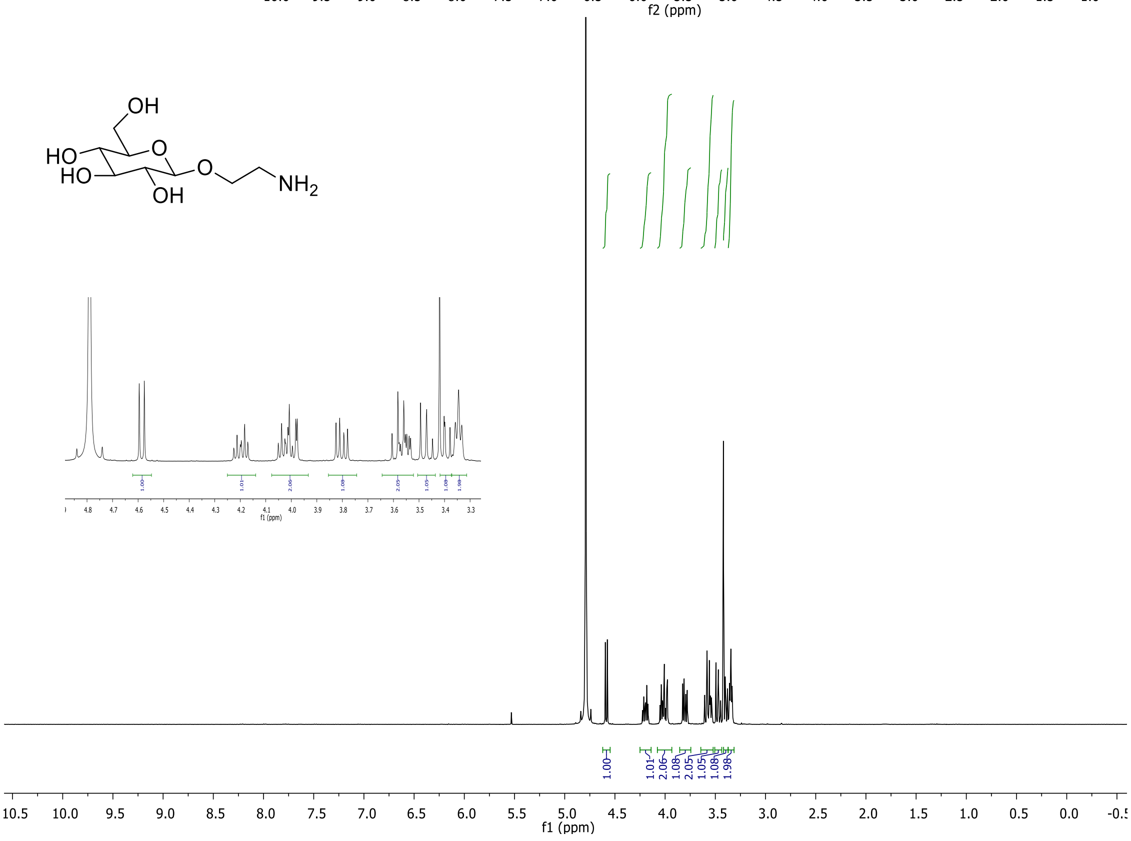

150

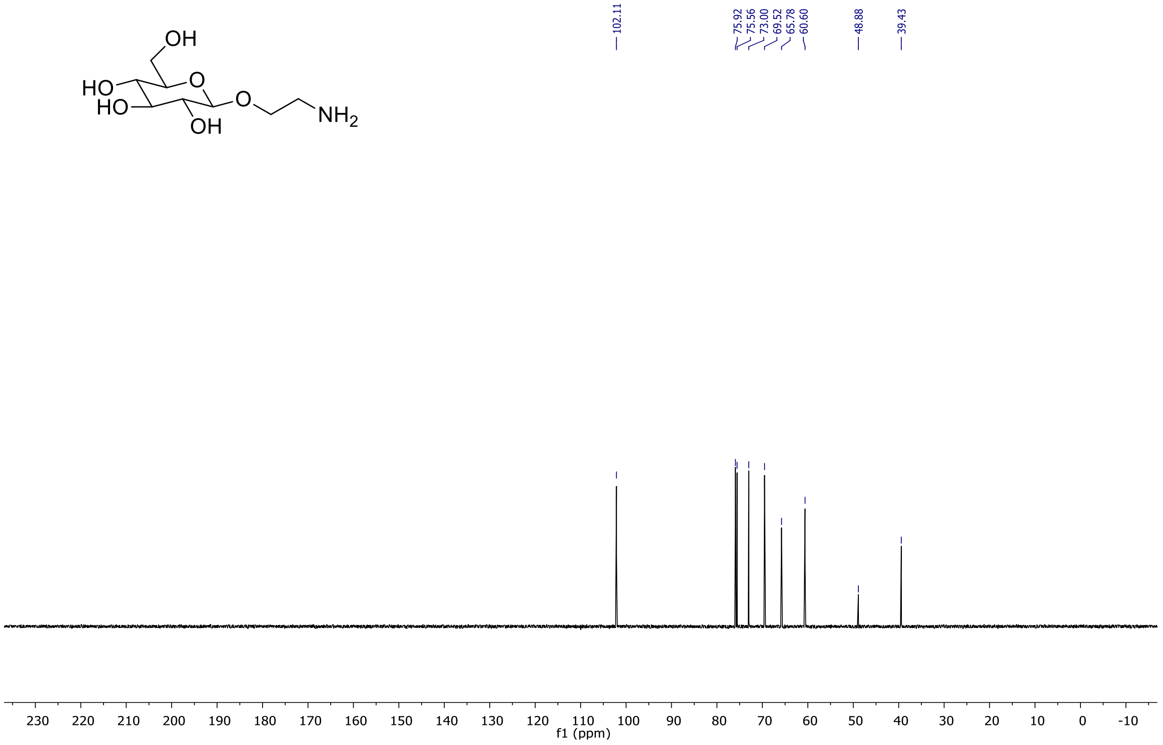

OH
HO
HO
O
O
OH
NH2
102.11
75.92
75.56
73.00
69.52
65.78
60.60
48.88
39.43
f1 (ppm)

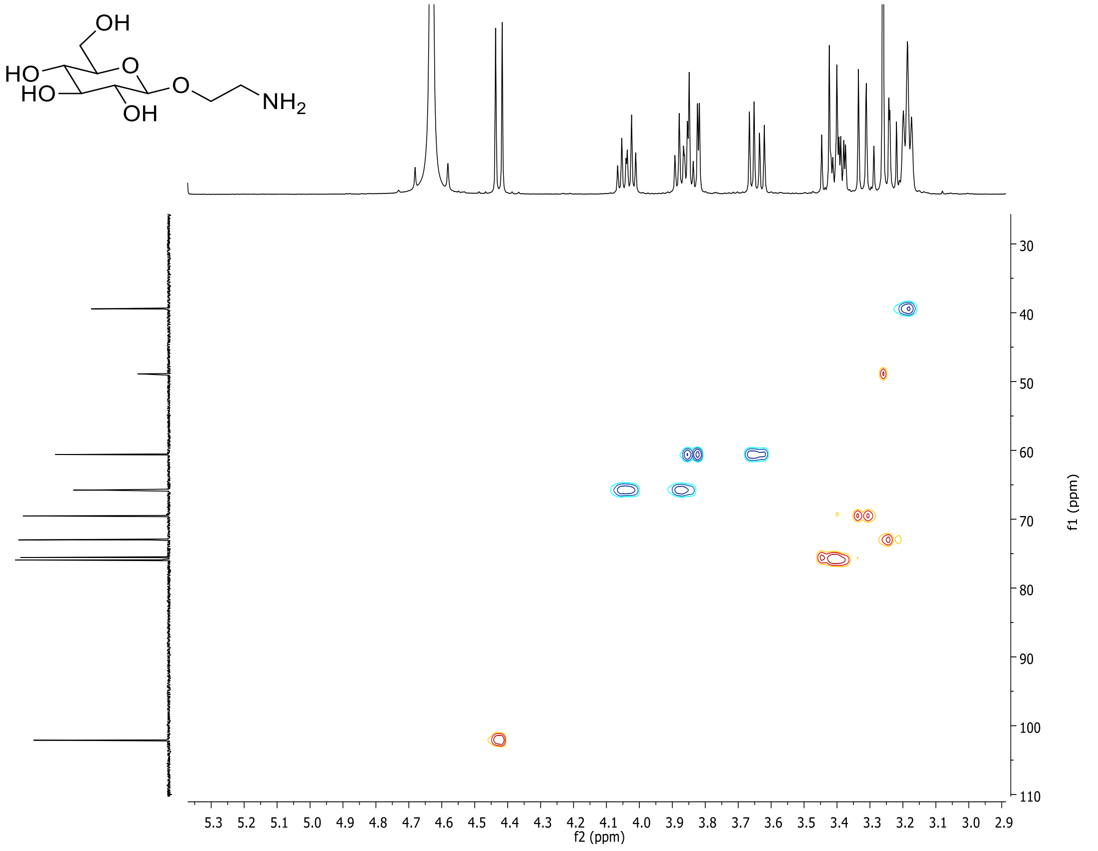

OH
HO
HO
O
O
OH
NH2
f2 (ppm)
f1 (ppm)

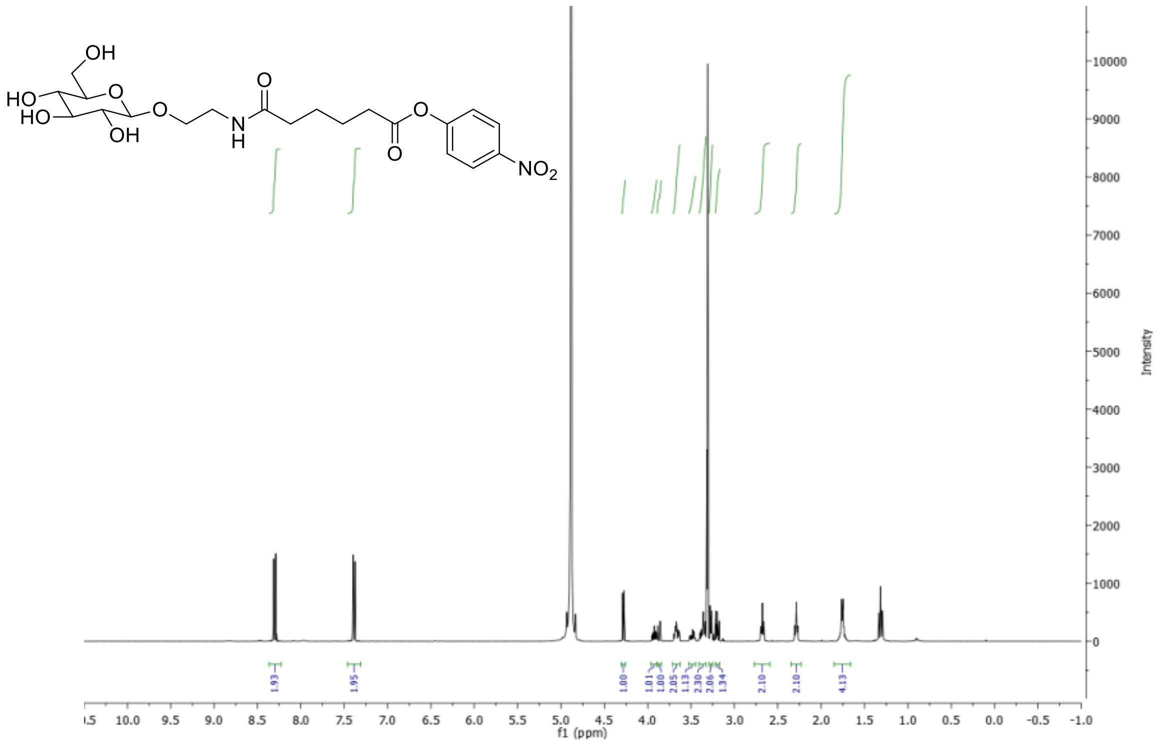
OH
HO
HO
OH
O
O
N
H
O
O
O
NO2
Intensity
f1 (ppm)
1.93
1.95
1.00
1.01
1.00
2.05
1.13
2.30
2.06
1.34
2.10
2.10
4.13

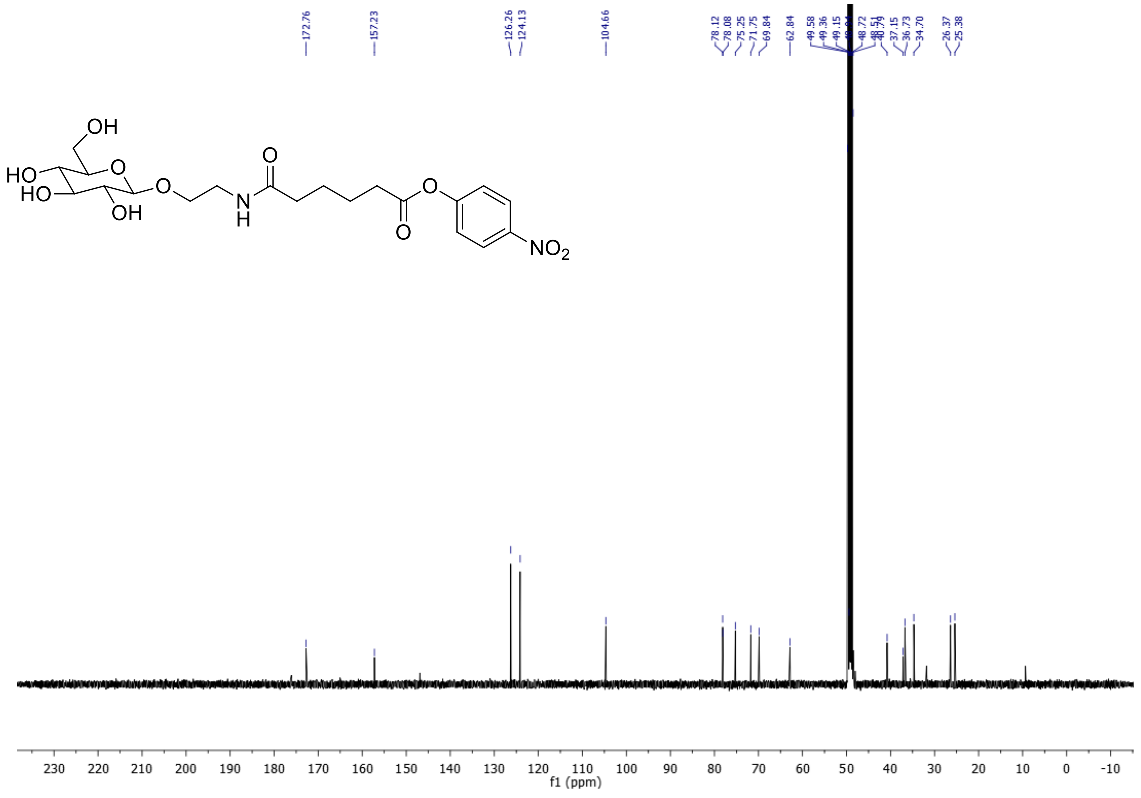
172.76
157.23
126.26
124.13
104.66
78.12
78.08
75.25
71.75
69.84
62.84
49.58
49.36
49.15
48.72
48.51
37.15
36.73
34.70
26.37
25.38
OH
HO
HO
OH
O
O
N
H
O
O
O
NO2
f1 (ppm)

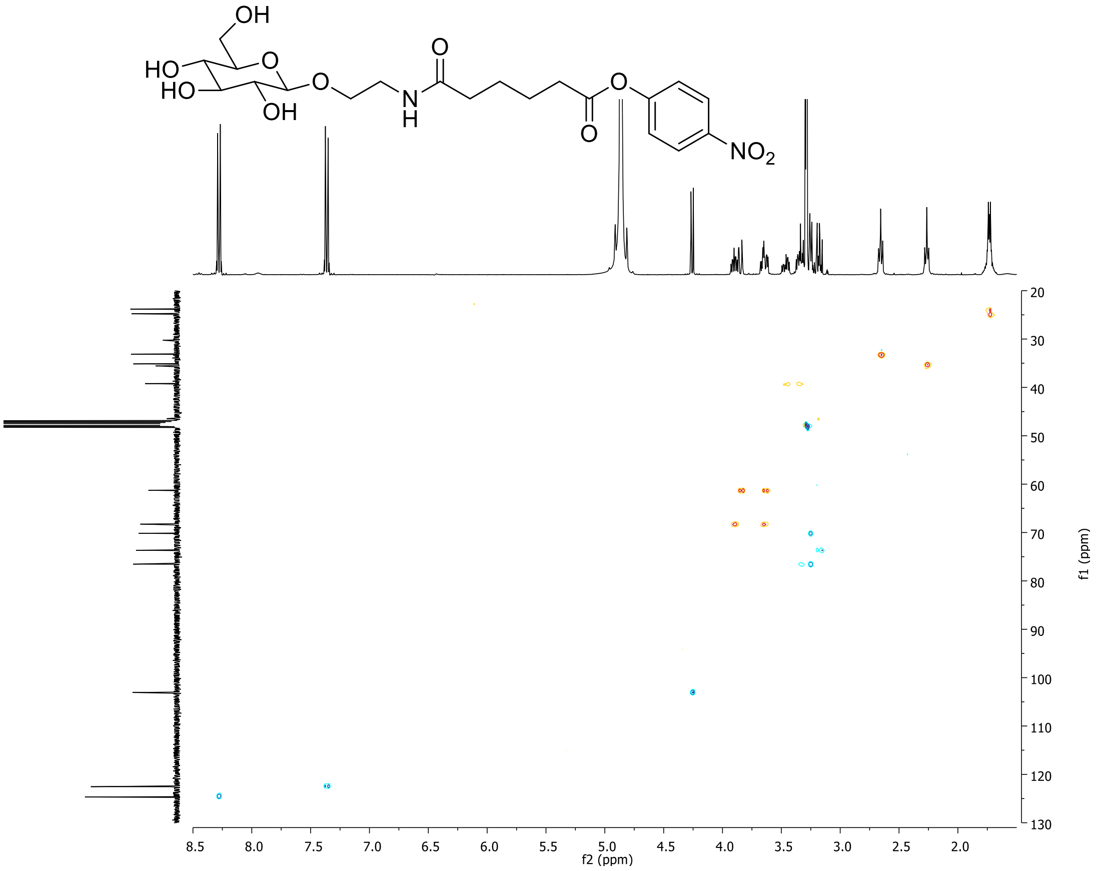
OH
HO
HO
O
OH
O
N
H
O
O
O
NO2
f2 (ppm)
f1 (ppm)
8.5 8.0 7.5 7.0 6.5 6.0 5.5 5.0 4.5 4.0 3.5 3.0 2.5 2.0
20 30 40 50 60 70 80 90 100 110 120 130

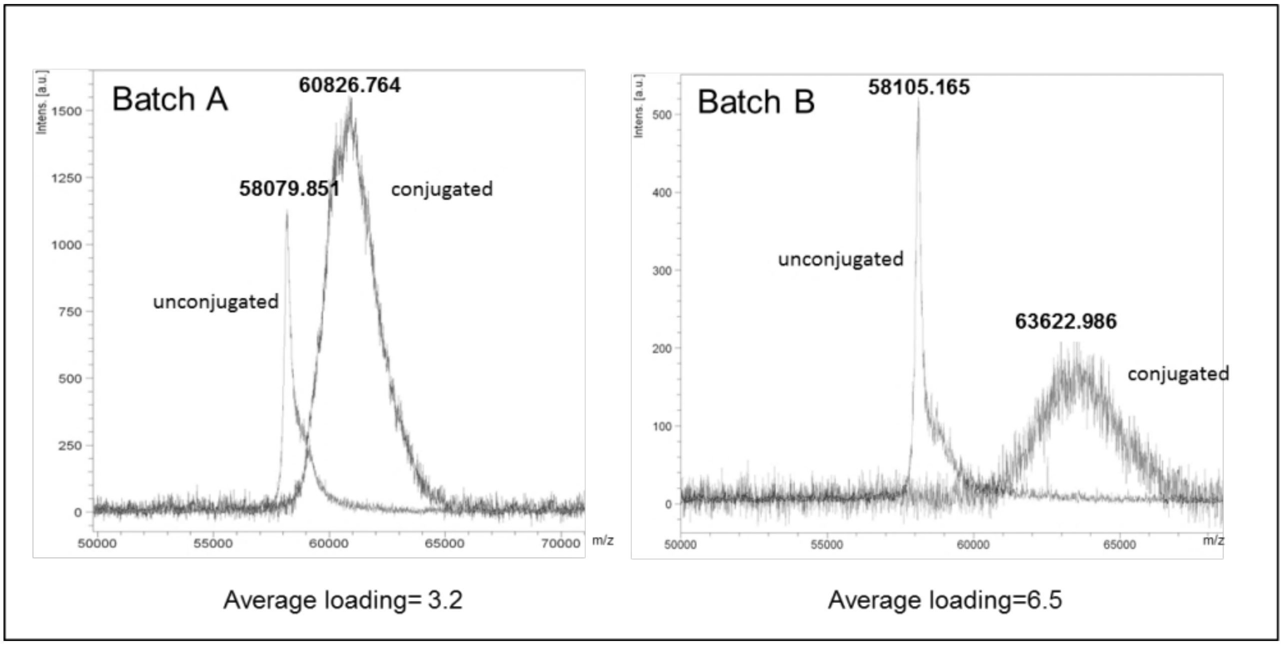

Supplementary Figure S8: MALDI-TOF-MS analyses of intact proteins from ST3 batch A and batch B. The mass shift indicated successful conjugation. The mass difference between unconjugated form and conjugated form was used to estimate an average loading.
